# The *Cissus quadrangularis* genome reveals its adaptive features in an arid habitat

**DOI:** 10.1093/hr/uhae038

**Published:** 2024-02-02

**Authors:** Qingyun Li, Yi Wang, Huimin Zhou, Yuanshuang Liu, Duncan Kiragu Gichuki, Yujun Hou, Jisen Zhang, Rishi Aryal, Guangwan Hu, Tao Wan, Sara Getachew Amenu, Robert Wahiti Gituru, Haiping Xin, Qingfeng Wang

**Affiliations:** CAS Key Laboratory of Plant Germplasm Enhancement and Specialty Agriculture, Core Botanical Gardens/Wuhan Botanical Garden, Chinese Academy of Sciences, Wuhan 430074, China; Sino-Africa Joint Research Center, Chinese Academy of Sciences, Wuhan 430074, China; University of Chinese Academy of Sciences, Beijing 100049, China; CAS Key Laboratory of Plant Resources, Institute of Botany, Chinese Academy of Science, Beijing 100093, China; CAS Key Laboratory of Plant Germplasm Enhancement and Specialty Agriculture, Core Botanical Gardens/Wuhan Botanical Garden, Chinese Academy of Sciences, Wuhan 430074, China; University of Chinese Academy of Sciences, Beijing 100049, China; CAS Key Laboratory of Plant Germplasm Enhancement and Specialty Agriculture, Core Botanical Gardens/Wuhan Botanical Garden, Chinese Academy of Sciences, Wuhan 430074, China; University of Chinese Academy of Sciences, Beijing 100049, China; CAS Key Laboratory of Plant Germplasm Enhancement and Specialty Agriculture, Core Botanical Gardens/Wuhan Botanical Garden, Chinese Academy of Sciences, Wuhan 430074, China; University of Chinese Academy of Sciences, Beijing 100049, China; CAS Key Laboratory of Plant Germplasm Enhancement and Specialty Agriculture, Core Botanical Gardens/Wuhan Botanical Garden, Chinese Academy of Sciences, Wuhan 430074, China; University of Chinese Academy of Sciences, Beijing 100049, China; Key Lab for Conservation and Utilization of Subtropical AgroBiological Resources and Guangxi Key Lab for Sugarcane Biology, Guangxi University, Nanning 530004, China; Department of Horticultural Science, North Carolina State University, Raleigh, NC 27695, USA; CAS Key Laboratory of Plant Germplasm Enhancement and Specialty Agriculture, Core Botanical Gardens/Wuhan Botanical Garden, Chinese Academy of Sciences, Wuhan 430074, China; Sino-Africa Joint Research Center, Chinese Academy of Sciences, Wuhan 430074, China; CAS Key Laboratory of Plant Germplasm Enhancement and Specialty Agriculture, Core Botanical Gardens/Wuhan Botanical Garden, Chinese Academy of Sciences, Wuhan 430074, China; Sino-Africa Joint Research Center, Chinese Academy of Sciences, Wuhan 430074, China; CAS Key Laboratory of Plant Germplasm Enhancement and Specialty Agriculture, Core Botanical Gardens/Wuhan Botanical Garden, Chinese Academy of Sciences, Wuhan 430074, China; Sino-Africa Joint Research Center, Chinese Academy of Sciences, Wuhan 430074, China; University of Chinese Academy of Sciences, Beijing 100049, China; Department of Botany, Jomo Kenyatta University of Agriculture and Technology, 62000-00200, Nairobi, Kenya; CAS Key Laboratory of Plant Germplasm Enhancement and Specialty Agriculture, Core Botanical Gardens/Wuhan Botanical Garden, Chinese Academy of Sciences, Wuhan 430074, China; Sino-Africa Joint Research Center, Chinese Academy of Sciences, Wuhan 430074, China; CAS Key Laboratory of Plant Germplasm Enhancement and Specialty Agriculture, Core Botanical Gardens/Wuhan Botanical Garden, Chinese Academy of Sciences, Wuhan 430074, China; Sino-Africa Joint Research Center, Chinese Academy of Sciences, Wuhan 430074, China

## Abstract

*Cissus quadrangularis* is a tetraploid species belonging to the Vitaceae family and is known for the Crassulacean acid metabolism (CAM) pathway in the succulent stem, while the leaves perform C_3_ photosynthesis. Here, we report a high-quality genome of *C. quadrangularis* comprising a total size of 679.2 Mb which was phased into two subgenomes. Genome annotation identified 51 857 protein-coding genes, while approximately 47.75% of the genome was composed of repetitive sequences. Gene expression ratios of two subgenomes demonstrated that the sub-A genome as the dominant subgenome played a vital role during the drought tolerance. Genome divergence analysis suggests that the tetraploidization event occurred around 8.9 million years ago. Transcriptome data revealed that pathways related to cutin, suberine, and wax metabolism were enriched in the stem during drought treatment, suggesting that these genes contributed to the drought adaption. Additionally, a subset of CAM-related genes displayed diurnal expression patterns in the succulent stems but not in leaves, indicating that stem-biased expression of existing genes contributed to the CAM evolution. Our findings provide insights into the mechanisms of drought adaptation and photosynthesis transition in plants.

## Introduction

*Cissus* is the largest genus of the Vitaceae family, with more than 300 species that are widely distributed in Africa, Asia, Australia, and the Americas [1, 2]. Many species in this genus have been used as traditional food and medicine for centuries [3, 4]. Africa has the highest species diversity and is thus regarded as an ancestral area of this genus [1]. *Cissus* species distributed in arid and semi-arid areas in Africa have evolved different strategies to withstand drought stress [5, 6]. Understanding the regulatory mechanism behind drought adaptive strategies in these species helps to understand the speciation within this genus and provides new insights for crop improvement.

Crassulacean acid metabolism (CAM) is a water-conserving photosynthetic pathway that has evolved convergently in different plants including those in the *Cissus* genus [7–9]. Although C_3_ plants possess all the enzymes necessary for CAM pathway, the critical difference between C_3_ and CAM lies on CO_2_ concentrating mechanism and temporal separation of a series of reactions [10, 11]. Phylogenetic analysis and multi-omics comparison have offered valuable insights into the origin and the transition from C_3_ to CAM, including duplication and the reconfiguration of core CAM enzymes in obligate CAM plants orchid (*Phalaenopsis equestris*), pineapple (*Ananas comosus*), and *Kalanchoe fedtschenkoi*, *Agave hybrid* NO.11648, respectively [12–15]. Furthermore, reprogramming of temporal network and rewiring of biochemistry metabolite were identified separately in facultative CAM plants *Sedum album* [16] and *Cymbidium mannii* [17], which provided new evidence of convergent evolution across CAM plants.

Polyploidization is widely occurring both in animals and plants. It contributes to species diversification through evolutionary innovations following chromosome rearrangements. The presence of multiple homologous chromosomes presents a major obstacle to generate a high quality genome assembly of a polyploid species. With the advent of long-read DNA sequencing technology such as PacBio, the genome assembly of many polyploid species is being developed. Polyploidization is also closely linked to agronomic traits in many crops, including improvement of fiber quality and tolerance in cotton [18], morphotypes in rapeseed [19], enhanced disease resistance in strawberry [20], and high seed oil content in peanut [21] and perilla [22].

**Figure 1:**
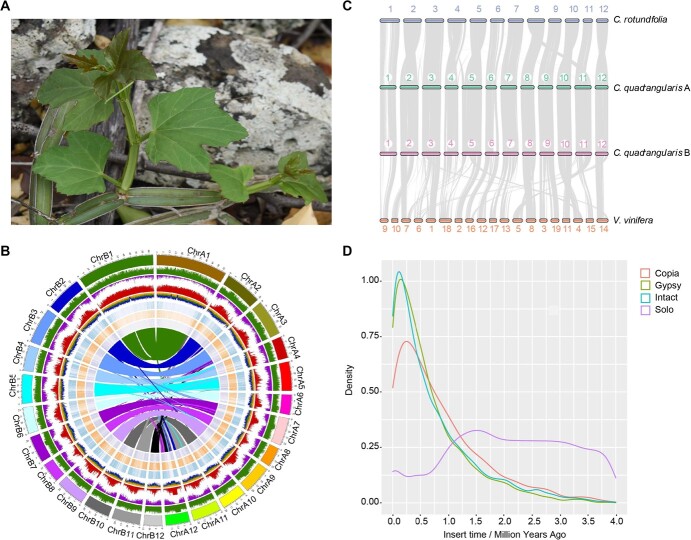
Morphology and genome evolution of *Cissus quadrangularis*. (**A**) The succulent, square stems and 3-lobed leaves of *C. quadrangularis*. (**B**) Distribution of genomic features of the *C. quadrangularis* genome. Each track shows GC content, gene density, repetitive sequence distribution, LTR density, and gene expression profiles in different tissues from outside to inside. The inner lines show the collinearity between two subgenomes. (**C**) The collinearity relationship among *C. quadrangularis*, *Cissus rotundifolia*, and *Vitis vinifera*. (**D**) The insertion time of different LTR types in *C. quadrangularis*.

*Cissus quadrangularis* is a sprawling perennial herb belonging to the *Cissus* genus (Fig. 1A). It is distributed throughout the tropical and subtropical regions and is native to India, Sri Lanka, Malaysia, Java, and Africa. The plant is used by local people as a medicinal herb [23, 24]. *C. quadrangularis* is known for its remarkable tolerance to drought stress [6]. Interestingly, leaves and stems of this plant display contrasting responses towards drought stress. The leaves only appear on new stems during the rainy season and are abscised under drought conditions [25]. Only succulent stems are left in the dry season, a typical feature of arid savanna.

Previously, studies on titratable acidity, gas exchange parameters, mesophyll succulence, and ^13^C/^12^C ratios have shown two modes of photosynthesis in *C. quadrangularis*, with CAM in stems and C_3_-like in leaves [26]. While organ-specific drought stress response and CAM regulations in *C. quadrangularis* is scientifically intriguing, lack of genome sequence limits the in-depth genetic research in this species. Our previous studies have revealed that *C. quadrangularis* is a tetraploid species (2n = 4× = 48) in contrast to diploid *Cissus rotundifilia* (2n = 2× = 24) [5, 27]. Sequencing a high quality genome will help to understand the genome divergence, subgenome evolution, and mechanism of drought adaption in *C. quadrangularis*.

In the present study, we performed a high-quality chromosome-level genome assembly, genome-wide transcriptomic analysis during drought periods, and a diel cycle from stems and leaves of tetraploid *C. quadrangularis*. We conducted a comprehensive comparison to infer preliminarily the cause and difference between two tissues in an individual displaying distinct phenotype under drought stress. In addition, we identified the core genes and putative regulatory factors involved in the CAM pathway in the stem of *C. quadrangularis*. Our efforts provide a valuable resource for comparative analysis and adaptability exploration, broadening our understanding of succulent plants thriving in arid environments.

## Results

### Genome assembly and annotation

*C. quadrangularis*, a perennial vine with succulent stems, is widely distributed in arid and semi-arid regions such as Africa and South Asia (Fig. 1A). The genome size of *C. quadrangularis* was estimated to be 689.49 Mb using flow cytometry analysis [27]. In this study, we calculated the genome size of *C. quadrangularis* ranged from 658.27 to 687.73 Mb using K-mer analysis on whole genome sequencing data, which is similar to the previous estimation (Fig. S1 and Table S1, see online supplementary material). K-mer frequency distribution plots for each K-mer size revealed a multimodal distribution with three clearly defined peaks (Fig. S1, see online supplementary material). The first two peaks at approximately 65 and 130 coverage correspond to the heterozygous and homozygous regions, while a distinct peak at approximately 260 coverage represents a tetraploid genomic composition. The heterozygosity ratio of *C. quadrangularis* was estimated to be 4.09% based on the 19 K-mer depth distribution, which was higher than that of the *C. rotundifolia* genome (1.19%).

*C. quadrangularis* draft genome was assembled from PacBio HiFi long reads, Illumina short reads, and Hi-C chromatin interaction reads (Table S2, see online supplementary material). The final genome assembly covered 679.24 Mb, comprising 670 scaffolds with an N50 size of 23.41 Mb. Approximately 90% of the assembled genome (613.34 Mb) could be anchored into 24 chromosomes (Tables S3 and S4, see online supplementary material). All centromere regions harboring centromere satellite arrays were identified on each chromosome. Using the plant-specific telomere repeat sequence as a query, we found 45 out of 48 telomeres in this assembly, 21 chromosomes have telomeres at both ends of them, while ChrB1, ChrB5, and ChrB9 have only one telomere (Fig. S2, see online supplementary material). The two largest chromosomes, ChrA1 (62.20 Mb) and ChrB1 (58.24 Mb), accounted for 17.88% of the assembled genome (Table S4, see online supplementary material). Synteny between sub-A and sub-B genomes also indicated that this was a tetraploid genome (Fig. 1B). Similarly, strong Hi-C signals between each chromosome pair among subgenomes further support two sets of homologous genome and high-quality genome assembly with accurately phased chromosomes (Fig. S3, see online supplementary material). Additionally, a high collinear relationship existed between the assembled genomes of *C. quadrangularis*, *C. rotundifolia*, and *Vitis vinifera*, suggesting the high completeness and quality of the assembled *C. quadrangularis* genome (Fig. 1C).

Mapping Illumina sequencing reads onto the assembled genome achieved a mapping rate of 99.75%, with less than 0.0027% error bases, indicating high sequence quality of the genome (Table S5, see online supplementary material). Similarly, Benchmarking Universal Single-Copy Orthologs (BUSCO) evaluation showed that 2308 (99.23%) out of 2326 core eukaryotic genes could be found in the *C. quadrangularis* assembly, including 2172 duplicated genes (93.38%), 121 single-copy genes (0.05%), and 15 fragmented genes (0.64%) (Table S6, see online supplementary material). Furthermore, the higher long-terminal repeat assembly index (LAI: 4.1–35.95, mean value of 14.77) also confirmed the good assembly contiguity (Fig. S4, see online supplementary material).

Gene model annotation of the genome was performed using three strategies, including *de novo* gene predictions, homology-based search, and transcriptome-based prediction of root, stem, and leaf. In total, 51 857 protein-coding genes were predicted, with an average gene length of 4208 bp and an exon length of 231 bp (Table S7, see online supplementary material). A total of 45 872 protein-coding genes (88.46%) were successfully annotated by six databases (NR, Swiss-Prot, Pfam, GO, KEGG, and 8ggnog) (Fig. S5 and Table S8, see online supplementary material), and 2572 of the protein-coding genes were predicted as transcription factors belonging to 56 gene families (Table S9, see online supplementary material). Additionally, a total of 11 615 non-coding RNAs were identified in *C. quadrangularis*, including 5229 rRNAs, 1684 snoRNAs, 251 microRNAs, and 4451 tRNAs (Table S10, see online supplementary material).

### Repeat content and recent LTR expansion in *C. quadrangularis* genome

In this genome, approximately 47.75% of the genome was identified as repetitive regions (Table S11, see online supplementary material). The majority of the transposable elements (TEs) were long-terminal repeat retrotransposons (LTRs), contributing 29.06% (Gypsy: 15.84%, Copia: 12.48%, and Unknown: 0.74%) of the total genome (Fig. S6A and Table S11, see online supplementary material). Approximately 64.99% (34 360 out of 52 866) of genes were affected by LTR elements in *C. quadrangularis*, which is slightly higher compared to *C. rotundifolia* (56.40%). Most LTR insertions occurred in the promoter and 3′-terminus regions of genes (Fig. S6B, see online supplementary material).

Transposable element burst, along with climate events, may induce new speciation events due to rapid genetic variation. We found that the LTR burst in *C. quadrangularis* occurred around 0.1 million years ago (Mya) (Fig. 1D), corresponding well to major biogeographic events of climate fluctuations in East Africa from drought to wet [28]. The solo to intact LTRs ratio (S/I ratio) was found to be slightly higher (S/I: 4.89, S: 40523, I: 8286) in the *C. quadrangularis* genome across LTR families than in *C. rotundifolia* (S/I: 4.37, S: 26170, I: 5987) and *V. vinifera* (S/I: 3.96, S: 20019, I: 5055). This higher S/I ratio implies that the *C. quadrangularis* genome underwent abundant ectopic recombination, which could decrease the activity of LTRs.

### Subgenome evolution and dominance analysis

Previous cytological analysis suggested that *C. quadrangularis* was a tetraploid species with 48 chromosomes (2n = 4×) [27]. The evaluation results from Genomescope 2.0 and Smudgeplot indicated that it is an allotetraploid [29] (Fig. S7, see online supplementary material). Two subgenomes in this tetraploid species were assigned based on the divergence time with the genome of *C. rotundifolia* (Fig. S8, see online supplementary material). The chromosomes with a closer relationship to *C. rotundifolia* were defined as the sub-A genome (chromosomes A1-A12), and the others were defined as the sub-B genome (chromosomes B1-B12). Thus, the genome size of A and B subgenomes was 302.37 Mb and 310.97 Mb, respectively (Table S4, see online supplementary material). BUSCO analyses, average nucleotide identity values, and collinear relationships of two subgenomes showed high-quality assembly and good collinear relationships (Fig. 1C; Fig. S9 and Tables S12 and S13, see online supplementary material).

Chromosome 1 pairs have relatively lower collinearity when compared with other chromosomes. Compared to Chr9 in *V. vinifera*, 39 959 616 bp of ChrA1 and 48 175 721 bp of ChrB1 in *C. quadrangularis* could not be found in collinear blocks (Fig. 1C). Whereas, compared to scaffold 1 in *C. rotundifolia*, 33 862 081 bp of ChrA1 and 39 501 792 bp of ChrB1 also could not be found in collinear blocks between the two *Cissus* species (Fig. S10, see online supplementary material), indicating that huge structural variation occurred between the three Vitis species during evolution. Among these regions, 1702 genes were uniquely distributed in *C. quadrangularis*, which were significantly enriched in amino acid metabolism, steroid biosynthesis, and bacterial toxins pathways (Fig. S11A, see online supplementary material). Only 720 genes were shared in two *Cissus* species, mainly enriched in flavonoid and phenylpropanoid biosynthesis, environmental adaptation, and plant-pathogen interaction pathways (Fig. S11B, see online supplementary material).

The syntenic and variation analysis of two subgenomes indicated that 6050 structural variations (SVs) were identified, including inversions (83), translocation (4859), and duplications (1108) (Fig. S12, see online supplementary material). According to the annotation, 3998 genes were located in the SVs affected regions, and these genes were mainly enriched in metabolism of terpenoids and polyketides, such as sesquiterpenoid and triterpenoid biosynthesis, flavonoid biosynthesis, stilbenoid, diarylheptanoid, and gingerol biosynthesis (Fig. S13, see online supplementary material). The genome formation analysis indicated that since an ancient polyploidization event (WGT-ϒ) occurred in *C. quadrangularis*, some chromosomes experienced a series of structural variations. Of the 12 chromosomes, Chr1, Chr4, Chr8, and Chr10 were relatively conserved whereas several chromosomal rearrangement events were observed in the remaining eight chromosomes (Fig. S14, see online supplementary material). The pattern of the chromosomal rearrangement was highly conserved between the two subgenomes, suggesting the formation of 12 chromosomes occurred before the speciation of *Cissus* and then entered the same genome by hybridization or other methods.

**Figure 2:**
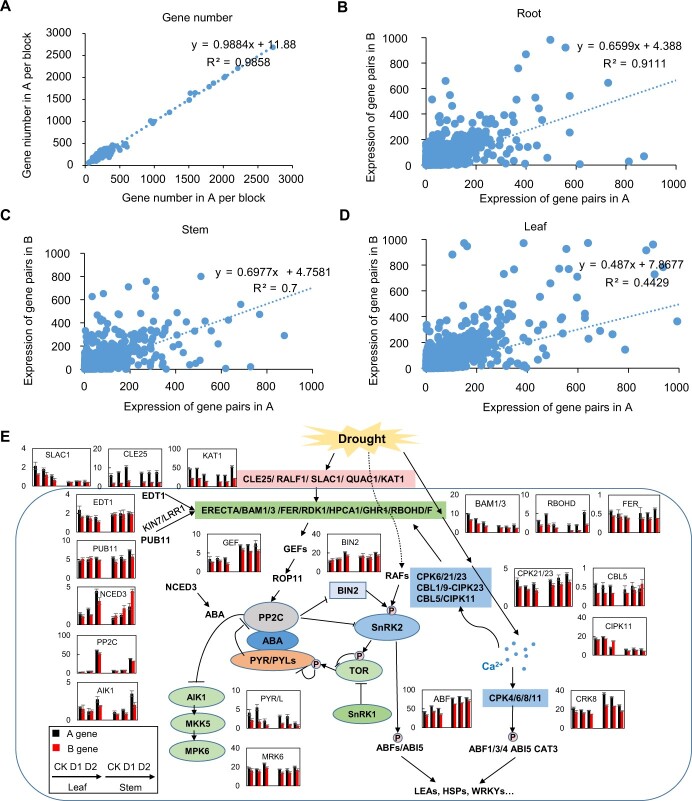
Gene number and expression of two subgenomes. (**A**) Gene number of two subgenomes in *Cissus quadrangularis*. Gene number in per collinear block between two subgenomes was used to plot and imitate to show the difference of gene content. (**B**–**D**) Gene expression in per gene pair between subgenomes in root, stem, leaf. The expressions of gene pairs were used to show the dominance of expression in *C. quadrangularis*. The collinear blocks and gene pairs were generated by MCScanX between subgenomes. (**E**) The expression of allelic genes associated drought signal pathway. A gene and B gene present allelic gene in sub-A genome and sub-A genome. CK, 60% SRWC; D1, 30% SRWC; D2, 10% SRWC.

Greater gene content and higher expression commonly occur in the dominant subgenome compared to other subgenomes following polyploidization [30, 31]. The gene and TE density showed no difference between the two subgenomes of *C. quadrangularis* (Fig. 1B and 2A; Fig. S15 and Table S14, see online supplementary material). Furthermore, the expression pattern of genes (FPKM > = 1) in different subgenomes was identified based on the transcriptome data. Similar numbers of expressed genes were identified from the two subgenomes in different samples. For example, a total of 23 618 genes were found to be expressed in roots (sub-A genome/sub-B genome: 10448 /10420), stems (9918/ 9903), and leaves (9440/9435). It was interesting that the expression level in the sub-A genome was higher than that in the sub-B genome, especially in the leaves (Fig. 2B–D), which supported sub-A genome as a dominant subgenome in this tetraploid. Thirty-five pairs of genes belonging to drought resistance signal pathway were identified in this assembly. Of them, 20 alleles were expressed higher in the sub-A genome (Fig. 2E), especially that the expressions of CLE25, KAT1, PYR/PYL were much higher in the sub-A genome than in the sub-B genomes. However, the expression levels of most genes in the sub-B genome were also increased and relatively high under drought stress.

### Evolution and gene expansion analysis of two subgenomes

**Figure 3:**
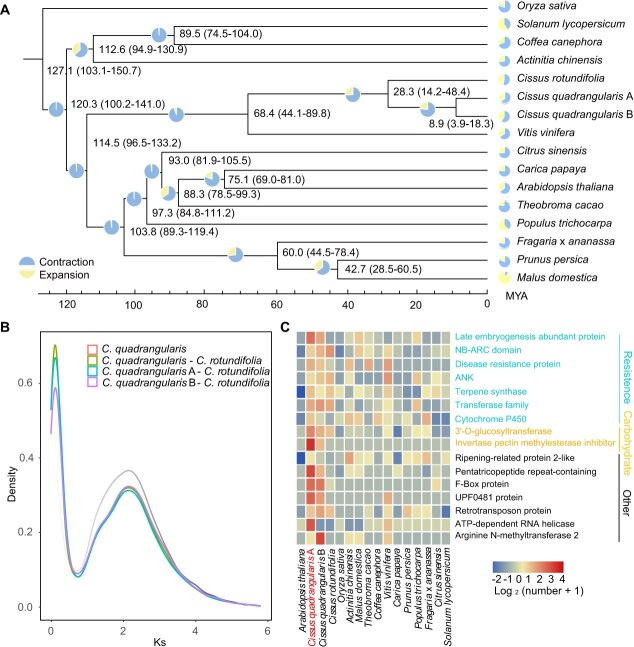
Evolutionary history and functional profiles of *Cissus quadrangularis* genome. (**A**) Phylogenetic tree showing gene family expansion/contraction analysis of two subgenomes of *C. quadrangularis* and selected 14 other representatives of eudicot plants. (**B**) The density distribution of *Ks* of *C. quadrangularis* vs *C. quadrangularis*, *C. quadrangularis* vs *Cissus rotundifolia*, *C. quadrangularis* A vs *C. rotundifolia*, and *C. quadrangularis* B vs *C. rotundifolia*. (**C**) Heat map showing gene families that have significantly increased paralogous numbers in *C. quadrangularis* sub-A genome compared with other angiosperms.

Comparative analysis of genome evolution was performed based on the genomes of 15 representative plant species, including the two subgenomes of *C. quadrangularis* (Table S15, see online supplementary material). A total of 21 638 gene families were identified among the selected species, and 95 single-copy gene families were selected to construct a maximum-likelihood phylogenetic tree (Fig. 3A). The results illustrated that *C. quadrangularis* and *C. rotundifolia* diverged approximately 28.3 million years ago (Mya). The divergence time between the *Cissus* and *Vitis* clades (about 68.41 Mya) was consistent with previously estimated divergence time [5]. The divergence time for the two *C. quadrangularis* subgenomes was estimated to be around 8.9 Mya (ranging from 3.9 to 18.3 Mya). Additionally, the distribution of synonymous substitutions per synonymous site (Ks) was used to evaluate duplication and speciation events in multiple species. A peak in the divergence of subgenomes with a Ks value of 0.08 indicated a divergence time of 4.49–5.61 Mya, which corresponds to the divergence time of the maximum likelihood analysis (Fig. 3B).

Among these selected species, the A and B subgenomes exhibited fewer expanded and contracted gene families than other species (Table S16, see online supplementary material). Only 18 and 19 gene families were significantly expanded in the two subgenomes. These significantly expanded gene families in both subgenomes involved pathways related to abiotic/biotic stress, carbohydrate metabolism, and other protein modifications (Fig. 3C and Fig. S16, see online supplementary material). Among the expanded genes were biotic and abiotic-related genes, including LEA (Late Embryogenesis Abundant) genes (20), NBS-LRRs (Nucleotide-Binding Site-Leucine-Rich Repeat) genes (13), and Cytochrome P450 genes (9). Moreover, genes related to 3'-O-glucosyltransferase and pectin methylesterase exhibited increased profiles, likely facilitating the modification and formation of pectin and polysaccharide components of the cell wall for succulent stems in *C. quadrangularis*. These results suggest that the expansion of the two subgenomes may contribute to the environmental adaptation of *C. quadrangularis*.

### Transcriptome analysis of drought stress adaptation

**Figure 4:**
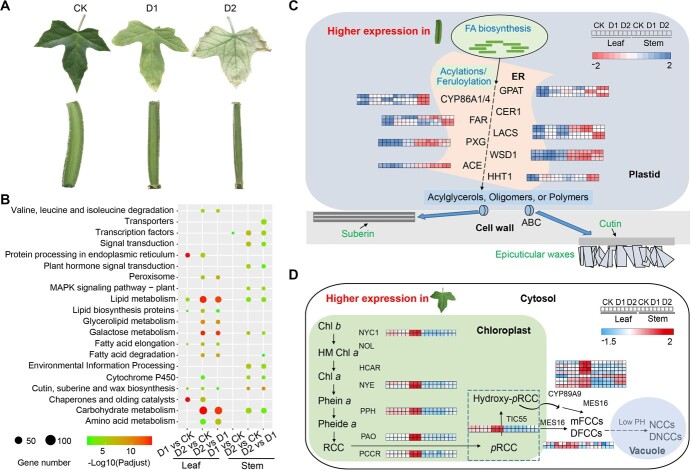
Different biological processes of leaves and stems toward the drought stress. (**A**) Phenotypic differences of leaves (upper) and stems (lower) in *Cissus quadrangularis* under drought treatments. (**B**) KEGG enrichment analyses of up-regulated differentially expressed genes (DEGs) among different comparisons for leaves and stems. L, leaves; S, stems. (**C**) Overview of the pathway of cutin, suberine, and wax biosynthesis. Various genes for acylations (acyl reduction, acyl activation, acyl elongation, etc.) and feruloylation are involved in this pathway. The ordering of genes shown does not imply the *in vivo* biosynthetic sequence for reactions. Heatmaps show relative expression of DEGs under different drought conditions of leaves and stems. (**D**) The overview of Chl degradation pathway. The steps of Chl catabolism occur in the chloroplast, cytosol, and vacuole through a series of gene modifications. Heatmaps show relative expression values of each gene under different drought conditions in leaves and stems.

We conducted a controlled drought experiment on *C. quadrangularis* and observed different drought responses in the leaves and stems of *C. quadrangularis*. The leaves gradually turned yellow and eventually withered, whereas the stems remained green though somewhat shrunken in size (Fig. 4A). To further investigate the transcriptional regulation of drought stress responses in *C. quadrangularis*, we performed whole-genome transcriptome analysis on the drought-treated leaves and stems. Transcriptome data were generated from two tissue types (leaves and stems) at normal (60% soil relative water content (SRWC), CK), moderate drought (30% SRWC, D1), and serious drought conditions (10% SRWC, D2). Each sample was sequenced in triplicate, resulting in a total of 18 RNA-seq datasets. Gene expression levels were analysed by aligning the reads to the *C. quadrangularis* genome. Principal component analysis and correlation analysis of gene expression (41 091 genes across 18 biological samples) showed good data quality within replicates (Fig. S17, see online supplementary material).

Differential gene expression analysis identified 9967 differentially expressed genes (DEGs). Leaves had more DEGs than stems in all comparisons. Relatively fewer DEGs of each tissue occurred in the D1 vs. CK comparison (Fig. S18A, see online supplementary material), suggesting less stress injury under 30% SRWC and extreme drought tolerance under 10% SRWC in *C. quadrangularis*. KEGG pathway analysis on the DEGs showed various metabolic pathways such as lipid metabolism, carbohydrate metabolism, galactose metabolism, and amino acid metabolism were activated in leaves. On the other hand, DEGs in stems mainly involved in cutin, suberine, and wax biosynthesis, cytochrome P450, signal transduction, and transcription factors pathways (Fig. 4B). Pathways such as ‘Photosynthesis’, ‘Plant hormone signal transduction’, and ‘Energy metabolism’ were enriched in downregulated DEGs in leaves (Fig. S18B, see online supplementary material). The expression patterns of DEGs involved in cutin, suberine, and wax biosynthesis were further shown in Fig. 4C. The expression of these genes in stems may have allowed stems to survive longer during dry environments. As a hallmark of leaf senescence, upregulated expression of DEGs associated with chlorophyll degradation was only observed in leaves (Fig. 4D), leading to the senescence and apoptosis of leaves.

### CAM photosynthesis in *C. quadrangularis*

**Figure 5:**
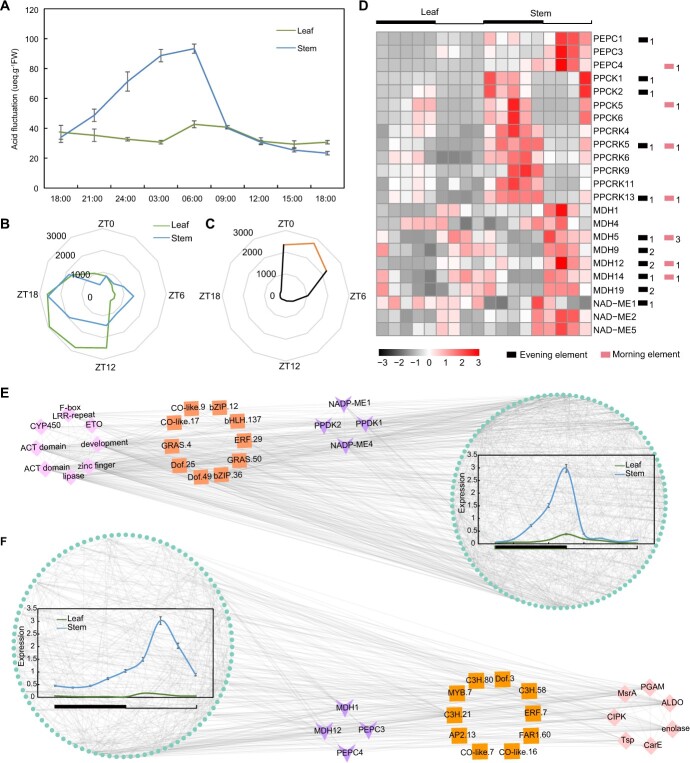
The CAM pathway in *Cissus quadrangularis*. (**A**) Diurnal variation of titratable acidity in C_3_-like leaves and CAM stems of *C. quadrangularis*. (**B**) Peak times of rhythmic transcripts across the 24-h diel cycle in two tissues. (**C**) Time of phase shift between leaves and stems. The full circle represents 24 h and each angle represents lagging time of gene expression in leaves compared to stems. Time points labeled orange were over 2000 shift genes. (**D**) Expression patterns and *cis*-regulatory elements of core CAM genes across the diurnal variation. The expression level of each gene is shown using the log10-transformed method. The numbers of evening element and morning element are shown in the 2-kb promoter region of each gene. (**E**–**F**) Two significant modules with opposite expression patterns in the two tissues were constructed by the WGCNA package. The square, diamond, and triangle represented transcription factor, hub-genes, and core CAM genes in respective modules.

To investigate the potential roles of transcriptional regulation in photosynthetic pathways, we performed titratable acid determination and transcriptomic analysis of the leaves and stems of *C. quadrangularis* in triplicate at every 3-hour interval over a 24-hour period (Fig. 5). The titratable acid abundance in leaves remained relatively stable at approximately 35 μeq g^−1^ FW during a diel cycle. However, the stems had a gradually increasing concentration of titratable acid at night, reaching the peak abundance at 06:00 (approximately 93 μeq g^−1^ FW), and began to fall at dawn (with the onset of light, Fig. 5A).

Rhythmic oscillation in the expression pattern of 11 305 (21.4%) genes in leaves and 10 322 (19.5%) genes in stems across the diel cycle was identified by JTK_CYCLE analysis (FDR-adjusted *P* < 0.01). To explore the phase shift of transcriptional regulation in leaves and stems of *C. quadrangularis*, two kinds of expressions were integrated and observed. Most rhythmic genes in leaves exhibited peak expression levels from the beginning of the night to midnight (zeitgeber time (ZT), ZT12-ZT20), while genes in stems showed peaks at noon (ZT6) and midnight (ZT16-ZT20, Fig. 5B). These rhythm genes with peak expression accounted for 76.7% and 50.7% of all rhythmic genes in leaves and stems, respectively. This phase shift pattern indicated that transcriptional regulation of circadian rhythm in stems occurred at or earlier than the modification in leaves. Compared to stems, the expression of genes in leaves was hysteretic, with approximately 48.5% of shift genes (10401) lagging by 2–4 hours, and 2337 genes had no time phase difference between the two tissues (Fig. 5C). Our results further imply that a weak leaf-stem phase shift pattern exists in *C. quadrangularis*.

To understand the transcriptional regulation of stem photosynthesis in plants, we focused our investigation on 85 core genes involved in CAM pathway. Although there was no significant expansion in these gene families (Table S17, see online supplementary material), several genes displayed apparent variations between daytime and nighttime compared to the C_3_-like leaves. These genes with distinct expression patterns included several key enzymes such as phosphoenolpyruvate carboxylases (PEPCs), malate dehydrogenases (MDHs), NAD-malic enzymes (NAD-MEs), phosphoenolpyruvate carboxylase kinases (PPCKs), and phosphoenolpyruvate carboxylase-related kinases (PPCKRs) (Fig. 5D; Fig. S19, see online supplementary material). Moreover, evening elements, combining evening-phased genes and morning elements, conferring dawn-phased expression [32], were presented in core CAM genes, suggesting the significance of these genes in CAM photosynthesis of stems.

Co-expression network analysis was performed on 6450 genes (12.2%) with rhythmically oscillated profiles. Out of the 18 modules identified, five showed significant circadian rhythmicity in stems (Figs S20 and S21, see online supplementary material). Among these modules, the MEdarkmagenta module included genes such as CYP450, Fbox-LRR repeat, ACT domain genes, and Dof, bZIP, and bHLH transcription factors (TFs) (Fig. 5E). Several biological processes, including cellular response to light stimulus, cellular response to radiation, and post-embryonic root morphogenesis, over-represented in this module (Table S18, see online supplementary material). Additionally, the MEdarkolivegreen module contained central hub genes like CIPK and Tsp, which cooperated with TFs such as Dof, ERF, and MYB to modulate diurnal gene expression (Fig. 5F). This module was enriched in biological processes related to ATP generation from ADP, glycolytic process, and purine nucleoside diphosphate metabolic process, which included genes like MDH1 and MDH12 (Table S18, see online supplementary material).

## Discussion

We have successfully sequenced and assembled the genome of tetraploid *C. quadrangularis* at the chromosome level. This high-quality genome assembly will significantly accelerate genomic evolution studies, not only within the *Cissus* genus but also within the broader Vitaceae family. Our assembly has revealed that *C. quadrangularis* is a tetraploid species consisting of two diploid subgenomes, shedding light on the origin of this species as an allotetraploid formed by two distinct *Cissus* species. This finding is consistent with previous results obtained from Illumina WGS datasets. The two subgenomes in *C. quadrangularis*, as well as the genome of *C. rotundifolia*, exhibit a smaller genome size, higher gene density, and lower transposable element (TE) content compared to *V. vinifera* [5, 33] (Table S19, see online supplementary material), indicating distinct genomic evolutionary patterns in different genera of the Vitaceae family. The higher ratio of syntenic (S) to non-syntenic (I) genes in both *Cissus* species suggests that the compact genomes in these two species may be due to the removal of proliferating LTRs [34].

Polyploid species, such as wheat [35], cotton [36], Brassica [37], and water caltrop [38], are known to undergo chromosomal rearrangements following polyploidization. Several recently formed allopolyploid genomes have exhibited megabase-scale structural variations, including translocations, rearrangements, and inversions, between their subgenomes [39, 40]. The concept of subgenome dominance is well established in allopolyploid species [40]; however, some allopolyploid plants, such as teff [41] and pumpkin [42] lack this feature. In the case of *C. quadrangularis*, we observed no chromosome-level rearrangements during the formation of its two subgenomes, but the sub-A genome displayed transcriptional dominance. The dominance gene analysis in the drought tolerance indicated that sub-A genome expressed more and maybe play a key role under drought treatments. However, the expression of the sub-B genome cannot be ignored in these processes. The results suggests that the combination of A and B subgenomes contributes to the high drought tolerance, sub-A genome is responsible for the drought tolerance, and sub-B genome is an effective complement against the drought.

Comparative genomic analysis has revealed similar patterns of gene family amplification (e.g., LEA, TPS) in *C. quadrangularis* and other succulent plants (Fig. 3C; Fig. S22, see online supplementary material). This suggests convergent evolution and adaptation among succulent plants to arid conditions. Similar findings were also observed in the genome of *C. rotundifolia*, indicating that the amplification of these gene families may have occurred before their differentiation [5]. The higher expression of several LEA genes during drought treatment (Fig. S23, see online supplementary material) provides additional evidence of their putative roles in coping with water deficiency in *C. quadrangularis*.

A shared and ancient whole genome duplication (WGD) event occurred in *C. quadrangularis*, coinciding with the timing of WGD events in *C. rotundifolia* and *V. vinifera*, representing a common whole genome triplication (WGT) event in all angiosperms [5, 33]. Additionally, a recent WGD event (tetraploidization) in *C. quadrangularis* may have driven the emergence of new features, such as CAM photosynthesis. However, it is worth noting that CAM photosynthesis can also be found in several species within the *Cissus* genus. Thus, explaining the origin of CAM in *C. quadrangularis* remains a significant challenge.

It is widely accepted that the CAM pathway has undergone multiple modifications in nearly all CAM plants. For instance, core CAM genes and their *cis*-regulatory elements have been identified in pineapple [13]. Rhythmic shifts and transcriptional regulation of photosynthesis-related genes under different conditions have been observed in the facultative CAM plant *S. album* [16]. Convergent evolution of stomatal movement and CAM genes has been noted in *K. fedtschenkoi* [14]. In the case of *C. quadrangularis*, we observed a tissue-specific diel expression pattern of core CAM genes. This implies the crucial role of transcriptional reorganization of existing genes in the evolution of CAM. However, *C. rotundifolia*, belonging to the same genus and exhibiting CAM in its leaves, shows a different group of diel-expressed genes. Further research is required to elucidate the evolution of CAM in *Cissus* species.

It is noteworthy that three dominant PEPCs notably express during the daytime in stems, which is consistent with previous enzyme activity studies in *C. quadrangularis* [43]. A similar expression pattern of PEPCs has been found in the leaves of *C. rotundifolia*. The regulation between the transcription and translation of these genes and their roles during CAM photosynthesis in *Cissus* species need to be further investigated.

In summary, the genome of *C. quadrangularis* provides new insights into the origin of the recent whole genome duplication event in *Cissus*. This will significantly benefit evolutionary studies not only within Vitaceae but also across all plants. It also suggests that plants can enhance their environmental adaptation through genome cooperation and dynamic expression of dominant genomes in different tissues. All these findings contribute to our understanding of allotetraploid origins and ecological adaptation.

## Materials and methods

### Plant materials

Plants of *C. quadrangularis*, introduced from Kenya, were cultivated in two different greenhouses at Wuhan Botanical Garden, CAS (Wuhan, Hubei Province, China). One greenhouse (hereafter referred to as the small greenhouse) is under controlled conditions (25°C and 16 h/8 h light/dark). Another greenhouse (hereafter referred to as big greenhouse) simulated the African climate under hot and dry conditions. The accession number of plants used in this study is MU0156.

### Library construction and sequencing

DNA of *C. quadrangularis* plants grown in the small greenhouse was extracted using the CTAB plant kit following a previously published protocol [44]. In brief, the leaves grown from stem-cutting plants growing for two months were prepared. Secondary metabolites were excluded by a washing step before CTAB extraction with the washing buffer including 50 mM Tris–HCL, 5 mM EDTA-Na_2_, 0.35 M D-sorbitol, 1% (w/v) polyvinyl pyrrolidone (PVP-K 30), and 1% 2-hydroxy-1-ethanethiol. ExpressTemplate Prep Kit 2.0 (PacBio, #100–938-900) was used to build a 15 kb insert library, and the constructed library was sequenced on the PacBio Sequel II platform Pacific Biosciences of California, Inc. (Menlo Park, California, USA) using the Circular Consensus Sequencing mode of single-molecule real-time sequencing technology. HiFi reads were generated by correcting and merging the sub-reads. Constructed libraries (150 bp paired-end) were sequenced on the Illumina Novaseq 6000 platform Illumina, Inc. (San Diego, California, USA).

Leaves from big greenhouse grown *C. quadrangularis* were collected for the Chromosome Confirmation Capture (HiC) libraries. The tissue was sent to Biomarker Technologies Corporation (Beijing, China) for HiC library construction and sequencing. HindIII was used to digest the DNA overnight. Then biotin labeling was added on sticky ends and digesting the proteins for cross-linked fragments was performed. These long and physical connections were further processed into chimeric fragments, subsequently used to create paired-end sequencing libraries. Finally, the Illumina HiSeq X Ten Illumina, Inc. (San Diego, California, USA) was used to sequence and produce 150-bp paired-end reads.

### Genome assembly

A hybrid approach that combined ~43× PacBio HiFi reads, ~150× Illumina sequencing reads, and ~196 × HiC reads was used for genome assembly. The genome size of *C. quadrangularis* was estimated using previously reported flow cytometry analysis and K-mer distribution of next-generation genome sequence data [27]. In order to exclude the effect of K-mer length, count frequencies for seven different kmer lengths (Kmer = 17, 19, 21, 25, 27, 29, 31) were produced by Jellyfish v2.2.4, and the kmer distribution histogram was plotted using GenomeScope 2.0 and Smudgeplot [29]. The heterozygosity and ploidy were also estimated from the histograms.

Hifiasm [45] was used to assemble the contigs of *C. quadrangularis* through the following three steps: correction of recognizable haplotype, construction of an assembly diagram, and generation of an assembly sequence, combining the HiFi reads, and HiC data with default parameters. Then, the Juicer software was used to map HiC reads onto the contig-level genome [46], and the mapped reads were used to cluster, order, and orient scaffolds onto chromosomes by 3D-DNA pineapple [47] with parameters: ‘--editor-saturation-centile 10 --editor-coarse-resolution 100000 --editor-coarse-region 400000 --editor-repeat-coverage 100’. The super-scaffolds were manually adjusted using Juicerbox [48] based on the heatmap and collinearity with the closely related species *C. rotundifolia* [5] (2n = 24, genome size = 371 Mb). The well-tuned genome was performed to order and orient again by 3D-DNA [46] script ‘run-asm-pipeline-post-review.sh’. Finally, all sequenced genomic data were used to fill gaps by FGAP v1.8.1 (http://www.bioinfo.ufpr.br/fgap/) and TGS-GapCloser v1.2.1 (https://github.com/BGI-Qingdao/TGS-GapCloser).

BUSCO v5.5.2 [49] was used to evaluate the genome completeness under genome model. BWA v0.7.15 was used to map Illumina reads onto the assembly [50]. LAI values generated by LTR_retriever v1.0.7 [51] were used to assess the quality of assembled genome. Telomeres were identified using tidk (https://github.com/tolkit/telomeric-identifier) with the plant-specific telomeric sequence ‘TTTAGGG’ as the query. The location of the centromere was detected by the Quartet toolkit [52].

### Identification of repetitive genomic elements

Transposable elements (TEs) in the allotetraploid *C. quadrangularis* genome were detected with homology-based searching. For homology-based detection, REPEATMASKER (v.3.3.0, http://www.repeatmasker.org/) was used to identify TEs against the REPBASE database (v20170127, https://www.girinst.org/) in the assembly. *De novo* TEs were detected by REPEATMASKER based on a *de novo* repeat library constructed by REPEATMODELER (v.1.0.4, http://www.repeatmasker.org/RepeatModeler.html), LTR_FINDER v.1.0.5 [53], and LTR_retriever v1.0.7 [50]. Solo-LTRs were identified using the script solo_finder.pl of LTR_retriever [50] with options: an alignment score >300, length >100 bp, and 80% coverage. The ratio of solo LTRs to intact LTRs (S/I) was calculated by the script solo_intact_ratio.pl of LTR_retriever [50]. Insertion time (T) of LTR retrotransposons was estimated by the formula: T = K/2r, the substitution rate ‘r’ was 1.3e-8 of per site per year and the genetic distance ‘K’ was calculated by the Kimura two-parameter model [54].

### Annotation of protein-coding genes

Homology-based, *de novo* and transcriptome-based prediction approaches were used to predict and annotate protein-coding genes. For the protein homology-based prediction, protein sequences of *Arabidopsis thaliana*, *V. vinifera*, *C. rotundifolia*, and *Oryza sativa* were retrieved from Ensembl Genome Browser (http://www.ensembl.org/index.html) and the website (http://www.grapeworld.cn/ggh/cis.html), and then the program of GenomeThreader (v1.7.1, https://genomethreader.org/) was used to predict gene structures and define gene models in *C. quadrangularis*. For the *de novo* prediction, three software suits including AUGUSTUS v3.3 [55], SNAP v2006.07.28 [56], and GeneMarkHMM v4.32 [57] were used to predict coding regions in the repeat-masked genome. For the transcriptome-based prediction, deep transcriptome sequencing of root, stem, and leaf tissues on Illumina platform (yielding 28.4 Gb datasets), was firstly mapped to the genome using TopHat v.2.2.1 [58], with mapping ratio of the reads ranging from 74.4% ~ 76.8%. The alignment bam files were then used as input for Trinity v2.2.0 [59] with option ‘—genome_guided_max_intron 10 000’ for genome-based transcript assembly. Moreover, the assembled transcripts were aligned to the assembly and filtered with the Program to Assemble Spliced Alignment v.2.0.2 [60] to detect likely protein-coding regions. Finally, gene models were obtained by EVIDENCEMODELER v.1.1.1 [61] to integrate all predicted gene models into a comprehensive non-redundant set of gene models.

Different databases were used to functional annotation of the *C. quadrangularis* genome. The protein sequences were aligned to Non-redundant (NR), SwitProt by BLASTP [62] with E-value cutoff of 1e-5. The sequences were also predicted in the Kyoto Encyclopedia of Genes and Genomes (KEGG) database to identify the set of KEGG Orthologs (KO) using the KEGG Automatic Annotation Server (KAAS) software [63]. Additionally, Eggnog-mapper v1.0.3 [64] was used to compare sequences against Eggnog database with ‘--database euk --dbtype hmmdb’. BLAST2GO pipeline [65] was used to annotate Gene Ontology (GO) for each gene. Family domains were extracted from Eggnog-mapper result. tRNA genes of *C. quadrangularis* genome were identified by tRNAscan-SE v1.4 software [66]. The rRNAs were detected using RNAmmer (v1.2, https://services.healthtech.dtu.dk/services/RNAmmer-1.2/) program using the parameters recommended for eukaryotic genome, and cmscan program of INFERNAL v1.1.2 [67] was employed to identify the miRNA and snRNA. Additionally, transcription factors (TFs) in *C. quadrangularis* assembly were predicted using PlantRegMap (http://plantregmap.gao-lab.org/).

### Synteny analysis and divergence time estimation

Based on the difference in divergence time between chromosome pairs and *C. rotundifolia*, we divided allotetraploid *C. quadrangularis* (4×, AABB) genome into the A and B subgenomes. Chromosomal structure variations were identified by MUMmer v4.0.0beta2 with default parameters (https://mummer.sourceforge.net/). Average nucleotide identity (ANI) for each chromosome pair was calculated by FASTANI v1.1 [68]. The synteny analysis was conducted and displayed by MCScanX [69] and Circos v0.69 [70]. Parallel alignments in proteins and DNA sequences were used by ParaAT v2.0 [71] with the parameters ‘-m mafft -f axt’. The nonsynonymous substitution rate (Ka) and synonymous substitution rate (Ks) values of the collinear orthologous gene pairs were calculated by KaKs_Calculator v2.0 [72]. The gene list of ancestral eudicot karyotype (AEK) was obtained from a previous report described by Murat *et al.* [73]. Gene pairs were obtained by MCScanX based on the BLASTP result of known AEK genes and genes in *C. quadrangularis* [62, 69].

For comparative phylogenetic analysis, orthologous genes from the two subgenomes and 14 other angiosperm species, including *C. rotundifolia*, *A. thaliana*, *O. sativa*, *V. vinifera*, *Actinidia chinensis*, *Coffea arabica*, *Solanum lycopersicum*, *Populus trichocarpa*, *Theobroma cacao*, *Carica papaya*, *Citrus sinensis*, *Fragaria ananassa*, *Malus domestica*, and *Prunus persica* were obtained using OrthoMCL v2.0 [74]. The MUSCLE (v.3.8.3, http://www.drive5.com/muscle/) was used to align single-copy orthologs (SCOs) extracted from above results and then SCOs concatenated into a super-gene alignment matrix. The high-quality aligned regions were obtained by GBLOCKS v.0.91b [75]. The trimmed alignment was further used to construct phylogenetic tree using RAXML-HPC v.8.2.8 [76] with parameters: Maximum Likelihood method, PROTGAMMALGX mode, and 1000 bootstrap replicates. Divergence times between species were predicted by the MCMCTREE program in PAML v.4.7a [77], with four fossil points including *C. arabica*-*S. lycopersicum* (72.4–104.9 Mya), *A. thaliana*-*C. papaya* (69.6–81.0 Mya), *P. persica*-*M. domestica* (9.0–79.7 Mya), and *F. ananassa*-*P. persica* (44.5–89.3 Mya), from TIMETREE5 (http://www.timetree.org/).

### Gene family expansion and contraction

The results of OrthoMCL v2.0 [74] across 15 species were applied to evaluate the gene family expansion and contraction using CAFE v4.2.1 [78] with default parameters. Six succulent plants including *C. quadrangularis*, *C. rotundifolia*, *A. comosus, K. fedtschenkoi*, *Kalanchoe laxiflora*, *Hylocereus undatus* and 13 non-succulent plants were used to analyse orthologs using the above method (Table S15, see online supplementary material). To explore the functional divergence, orthologs with *P*-values of less than 0.05 were plotted by ggplot2 (https://ggplot2.tidyverse.org/).

### RNA-seq data generation and gene expression analysis

RNA sequencing (RNA-seq) data were used to improve genome annotation, compare gene expression differentiation of leaves and stems under drought stress, and search for key genes in th CAM pathway. To improve the functional annotation, roots, stems, and leaves of *C. quadrangularis* from the big greenhouse were collected for RNA extraction. For drought treatments in *C. quadrangularis*, nine stem-cutting plants of *C. quadrangularis* were grown for four months in the small greenhouse under normal conditions with nutrient soil (soil/vermiculite 2:1) and watered every three days. On 13 July 2022, the biomass of each plant was weighed and recorded. To simulate the African growth environment (higher temperature and lower humidity), all plants had their soil matrix changed to 500 g volcanic stone. They were sequentially cultured in the small greenhouse with watering once a day for 10 days, and then transferred to the big greenhouse for eleven days. To adapt to the environment of the big greenhouse (35°C/20°C and 20%RH/50%RH in day/night), we watered nine plants for the first three days and then stopped watering. To obverse the SRWC, we weighed the plants and recorded it from the fourth to the 11th day, twice daily. The second and third leaves and the intermediate stem for every three plants were harvested when SRWC of plant, respectively, reached 60%, 30%, and 10%.

For the CAM study of leaves and stems in *C. quadrangularis*, a total of 54 stem cuttings grown for four months in the small greenhouse were wrapped with plastic wrap to prevent water loss and then transferred to the artificial light incubator (25°C, 30%RH, 20000 h) for five days. These plants adapted to the environment in the first three days. The second and third leaves and the fourth stems per individual were harvested for RNA extraction every 3 hours from 18:00 on 19 September 2022 to 18:00 on 20 September 2022. Each experiment had three biological replicates. All samples of plants were immediately kept at −80°C for RNA extraction and titratable acid determination.

RNA from all samples was extracted using the BioTeke Kit (Wuxi, Jiangsu, www.bioteke.cn) following its standard protocol. The RNA quality was evaluated using the Fragment Analyzer (AATI, Standard Sensitivity RNA Analysis Kit (15 nt), DNF-471). Then libraries were built using the TruSeq RNA Library Kit (Illumina, NEB) and sequenced on a DNBSeq platform Mgi Tech Co., Ltd. (Shenzhen, Guangdong, China)with 150 bp paired-end reads. For transcriptome analysis, the raw data was assessed by FastQC (https://www.bioinformatics.babraham.ac.uk/projects/fastqc/). Quality control of RNA-seq including removing and trimming reads were performed by Trimmomatic-0.36.jar v2.2.1 [79]. Clean reads were then mapped to *C. quadrangularis* genome using TopHat v2.2.1 [56] with default parameters. Mapped reads were used to calculate the expression levels by Cufflinks v2.2.1 [58]. Correlation and PCA were employed by ‘cor’ and ‘prcomp’ functions. The gene satisfied with two requirments, |Log2(Fold Change)| > 1 and false discovery rate (FDR) adjusted *P* value <0.05 between two groups, was determined as differentially expressed genes (DEG). The functional enrichment analysis was implemented by agriGO (http://bioinfo.cau.edu.cn/agriGO/) and TBtools (https://github.com/CJ-Chen/TBtools).

### Diurnal and co-expression network analysis

All transcripts from stems and leaves over a diel cycle in *C. quadrangularis* were firstly filtered according to the average FPKM >1. The filtered transcripts were then used to determine the rhythmicity by JTK_CYCLE algorithm [80]. The period parameter was set to 24 h, and the replicate was set to 3. The cycle data were defined by cut score of FDR *P* < 0.01. Finally, co-expression networks of rhythmic transcripts from leaves and stems in *C. quadrangularis* (54 samples) were built by the WGCNA package with the following parameters: powers = 12, minModuleSize = 30.

### Titratable acid determination

Titratable acidity abundances in stems and leaves of *C. quadrangularis* plants, grown in an artificial illumination incubator (12 h/12 h: dark/light, 25°C) at a 3-hour interval over a diel cycle were determined according to the method described by Chen and Black [81] with minor modifications. Firstly, a total of 0.5 g of frozen tissue per sample were weighed and cut into pieces, and placed in a 50 mL centrifuge tube. Then, we added 10 mL of carbon dioxide-free distilled water to the tube, and the mixture of the tube was boiled. The mixture was cooled to room temperature and filtered after 30 minutes boiling. The above process of adding water, boiling, and filtering was repeated one more time on the filtered residues and the filtrates obtained in the two runs were combined together. Finally, the titratable acid in filtrates was titrated to a pH of 8.3 using a 0.01 mol · L ^−1^ NaOH solution and the solution volumes were recorded. Titratable acid abundances in stems and leaves were calculated and then showed in microequivalents per gram fresh weight (μeq g^−1^ FW).

### Identification of gene families

The genomic resources of in *A. comosus*, *V. vinifera* (PN40024), *O. sativa*, and *Zea mays* were obtained from the Phytozome database (https://phytozome-next.jgi.doe.gov/). The genome dataset of *P. equestris* was downloaded from the NCBI National Center for Biotechnology Information (https://www.ncbi.nlm.nih.gov/) and genome sequences of *C. rotundifolia* were derived from www.grapeworld.cn/ggh/cis.html. The list of core CAM genes was obtained from the supplemental file of *C. rotundifolia* and *A. comosus* genomes. The protein sequences of core CAM families from the above species were extracted by the Perl program, used as queries to search against *C. quadrangularis* genome-wide sequences by BLASTP v2.6.0 [62] with alignment length >100 bp and E-value <1e-5, and the Hidden Markov models (HMM) for each family were built using ‘hmmbuild’ of HMMER (v2.19, https://www.ebi.ac.uk/Tools/hmmer/) with default parameters. The ‘hmmersearch’ program was used to screen the possible genes with HMM, and the results from BLASTP and HMMER were merged together as the input for domain visualization. After visual domain validation using CD-search (https://www.ncbi.nlm.nih.gov/cdd) on NCBI, we defined the putative candidates for each gene family. The identification of the LEA gene family was done using the same method described above for the CAM gene family.

### Enrichment analysis of *cis*-regulatory elements

The morning element (CCACAC), and the evening element (AAAATATCT) in promoter regions (2 kb) on core CAM genes were searched and screened using Find Individual Motif Occurrences (v5.5.1, https://meme-suite.org/meme/tools/fimo) with the an E-value cutoff of 1e-4 and PlantCARE software (http://bioinformatics.psb.ugent.be/webtools/plantcare/html/).

## Supplementary Material

Web_Material_uhae038

## Data Availability

All data used and generated in this study have been deposited on the National Genomics Data Center (NGDC, https://ngdc.cncb.ac.cn/) with the project number PRJCA020539. All data is available from the corresponding author upon reasonable request.
