# Supplementary material for: The Cissus quadrangularis genome reveals its adaptive features in an arid habitat
Source: Hortic Res. 2024 Feb 2;11(4):uhae038. doi: 10.1093/hr/uhae038 (PMC11001597; doi:10.1093/hr/uhae038)
Supplement: Web_Material_uhae038 [file web_material_uhae038.zip › 3.1. Supplemental Figure 1-10.docx]

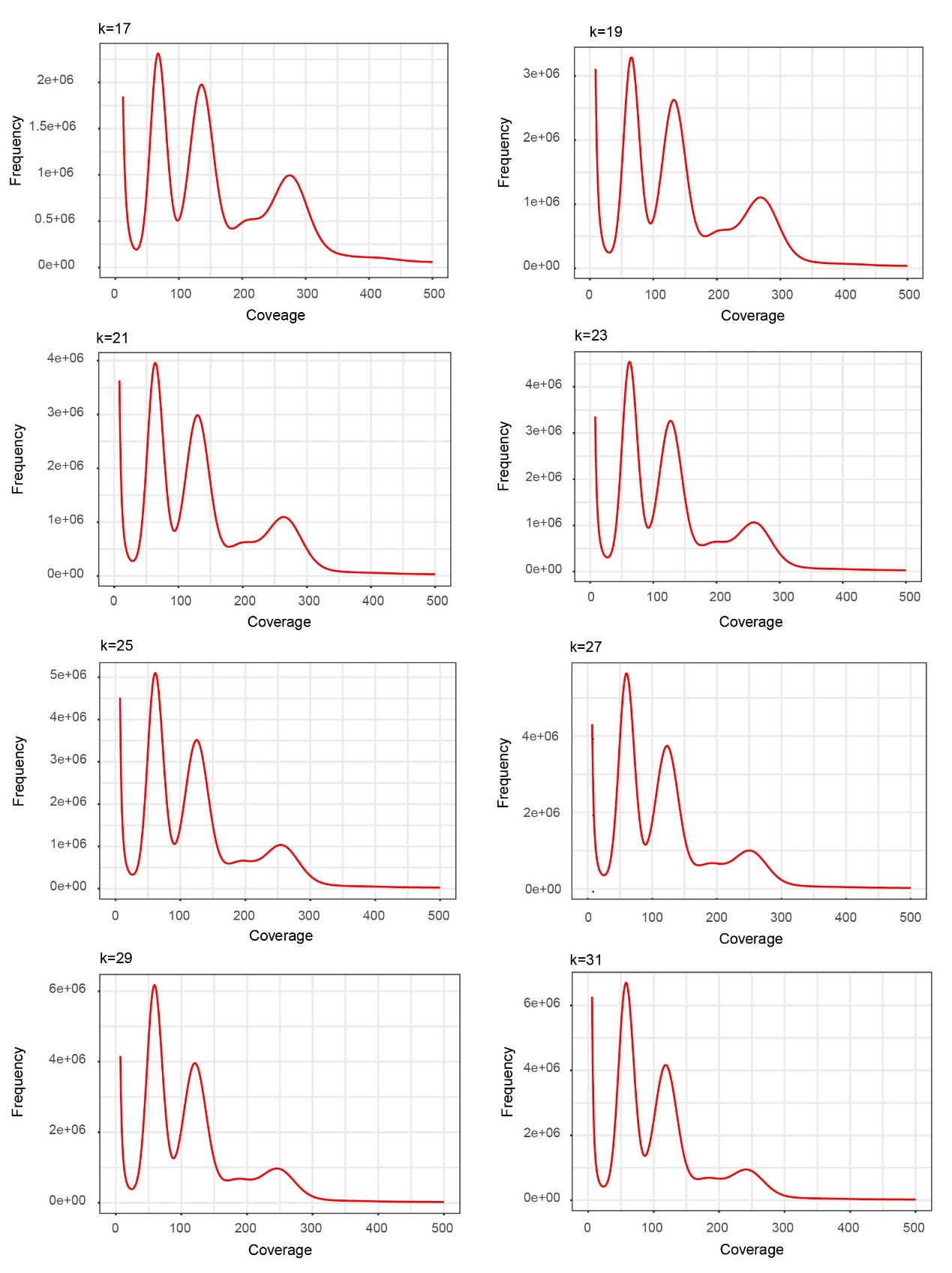


Figure S1. Comprehensive K-mer based genome size estimates. K = 17, 19, 21, 23, 25, 27, 29, 31 and 71 count histograms of Illumina paired-end sequence data for the tetraploid *Cissus.* *quadrangularis* are given.


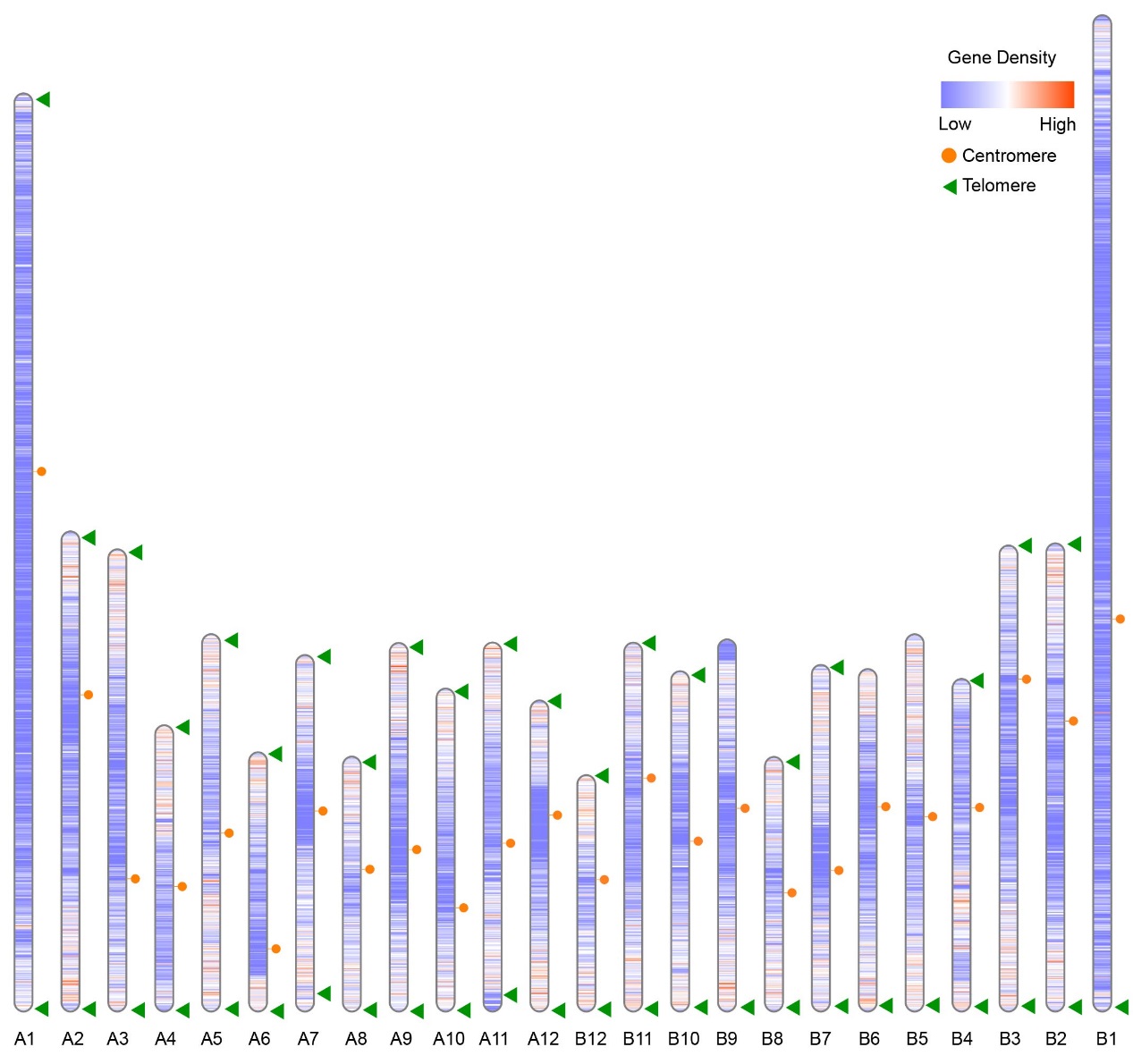


Figure S2. Visualization plant-specific seven-base telomere repeat sequence in assembly.


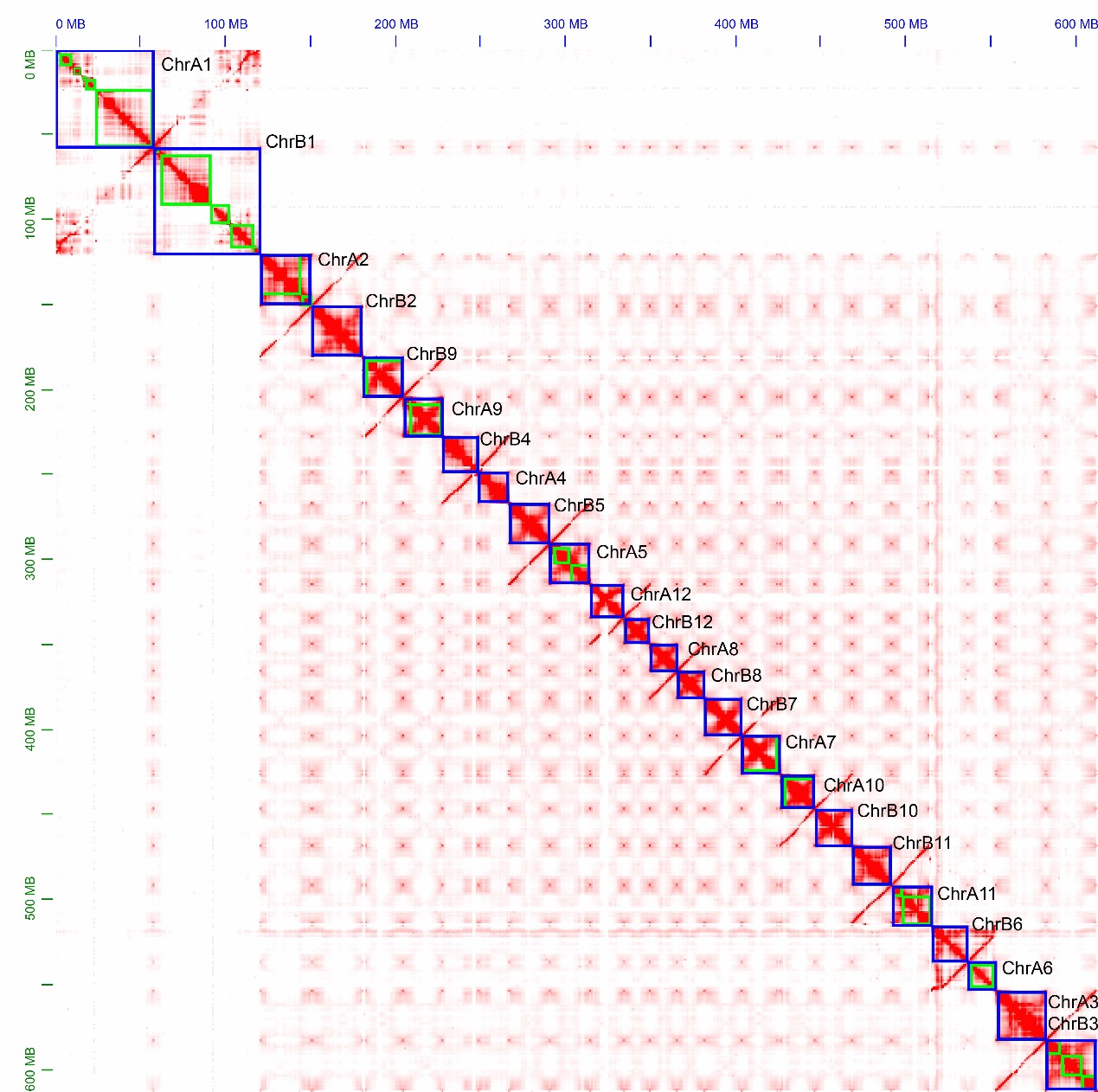


Figure S3. Hi-C-based clustering of the *C. quadrangularis* genome. Heat map show the density of Hi-C interactions between contigs. Distinct chromosomes are highlighted by blue boxes.


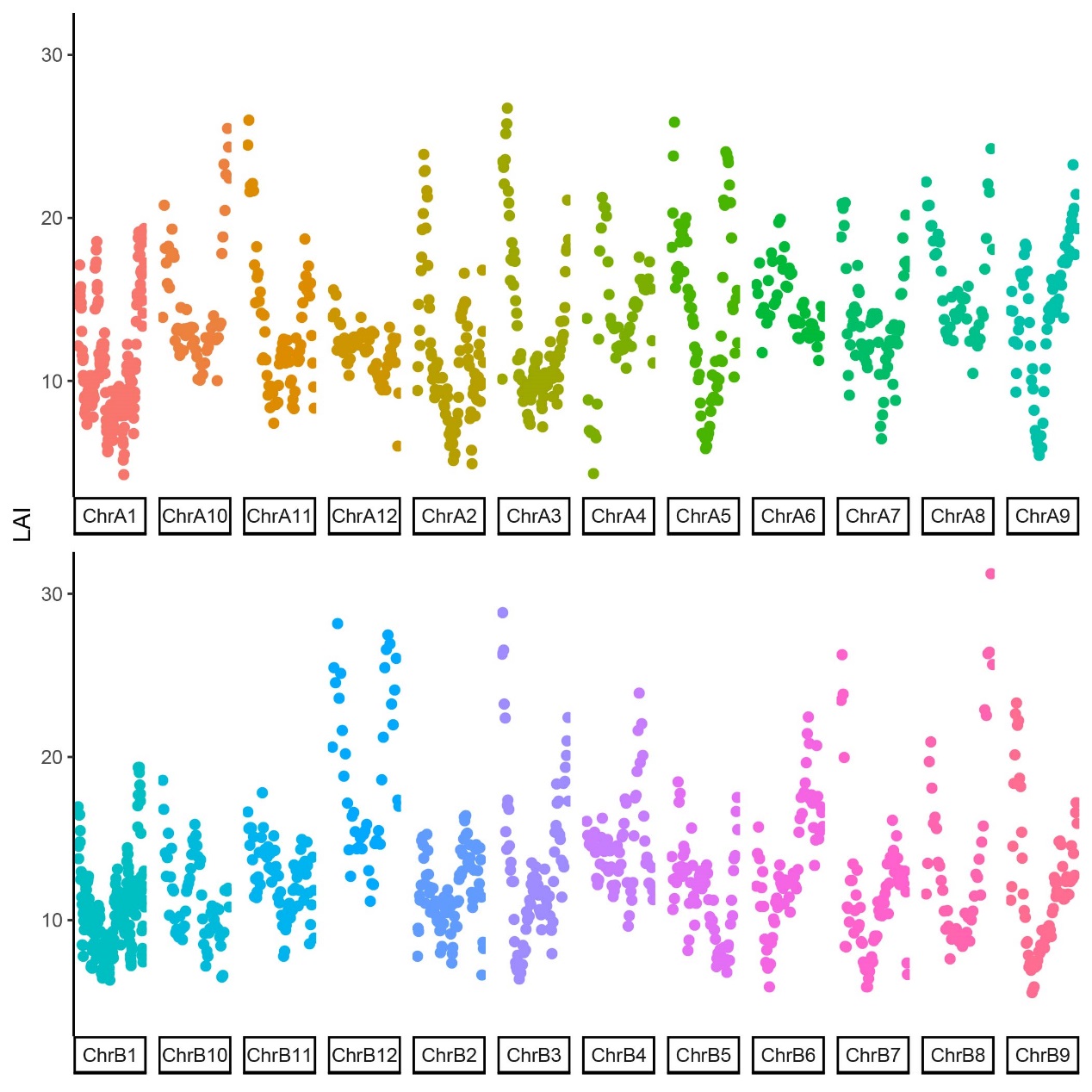


Figure S4. Long terminal repeat assembly index assessment of *C. quadrangularis* assembly.


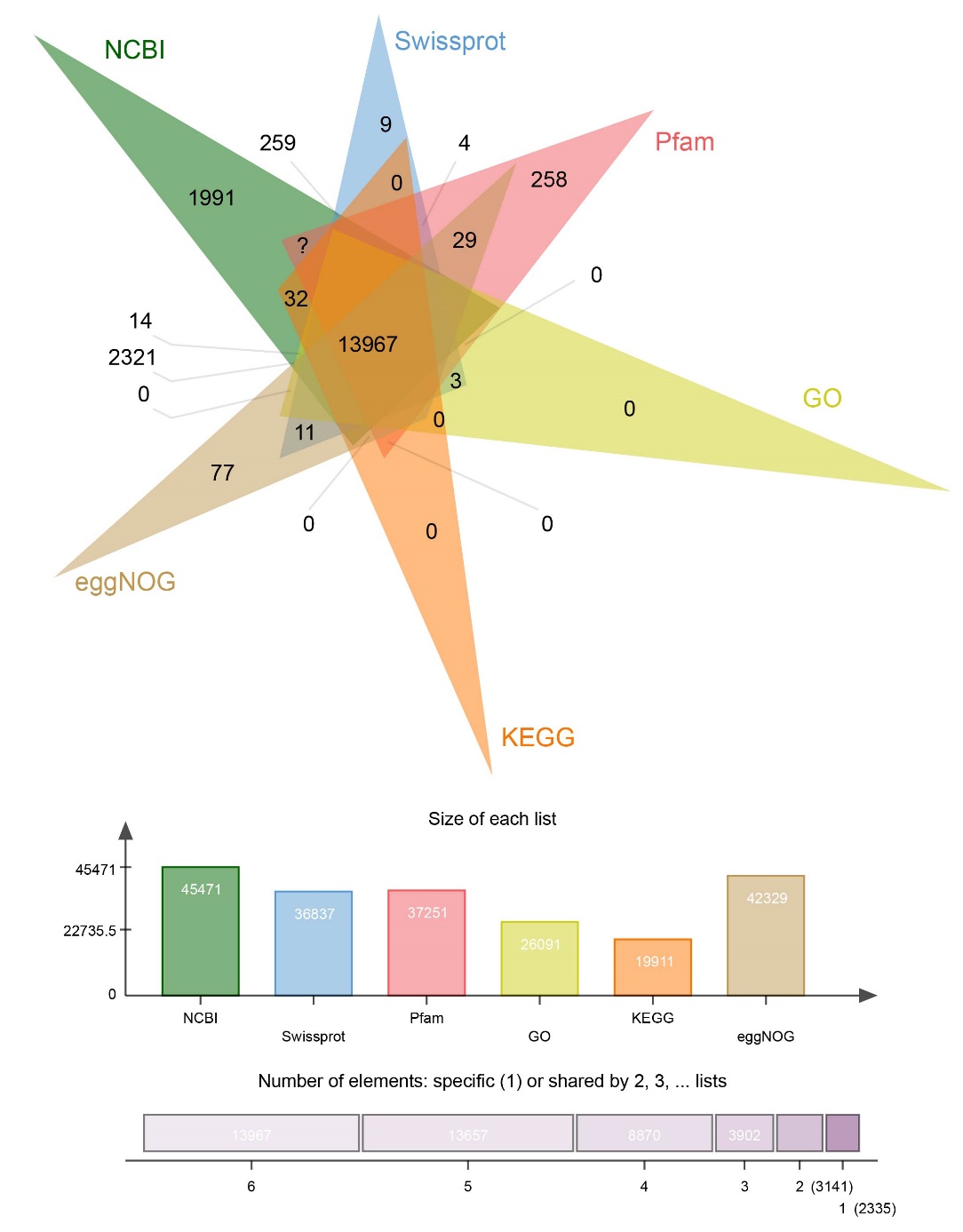


Figure S5. Functional annotation of *C. quadrangularis* genome in NR, Swiss-Prot, Pfam, GO, KEGG and eggNOG databases.


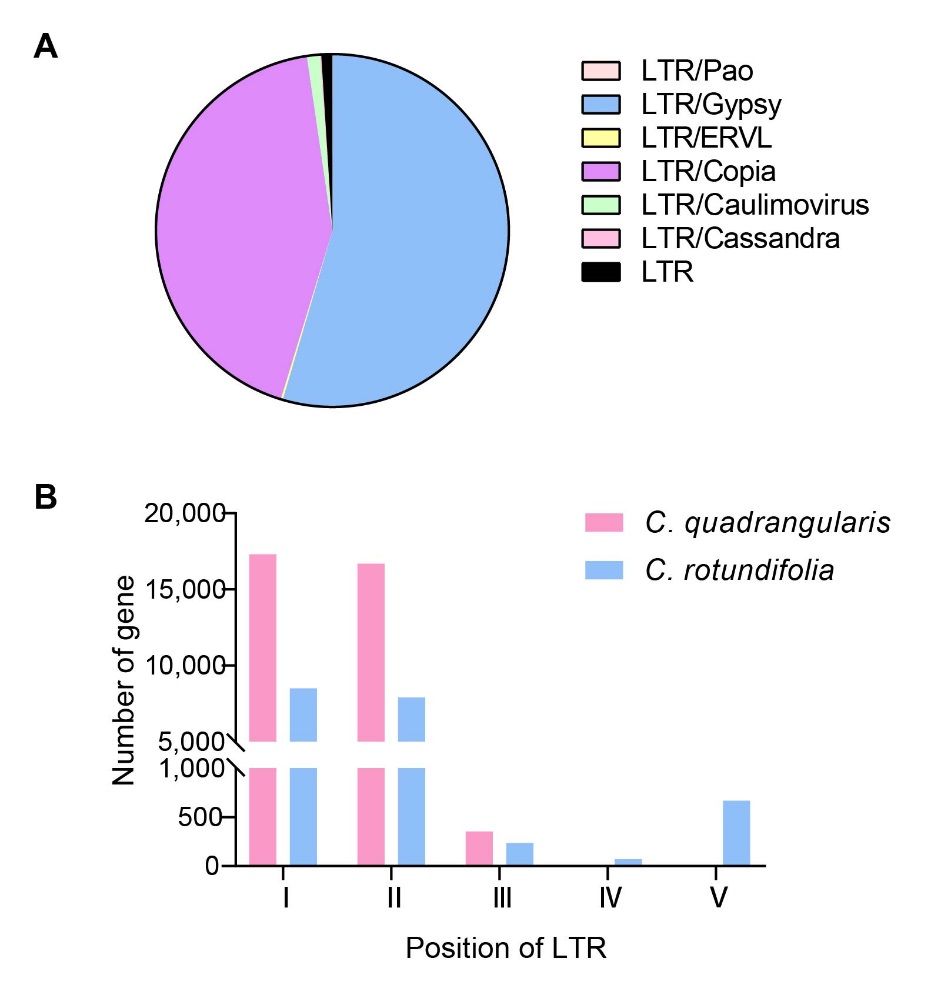


Figure S6. LTR insertions to the *Cissus* genomes. A, Different types LTR proportion in *C. quadrangularis*. B, Numbers of genes inserted by LTR retrotransposons. Column (I) shows the number of genes whose promoter region was inserted by LTR; Column (II) indicates the number of genes whose 3’-terminus region was inserted by LTR; Column (III) indicates the number of genes whose encoding region was inserted by LTR; Column (IV) shows the number of genes that located within the LTR, and column (V) indicates the number of genes with sequences that partially overlap with the LTR.


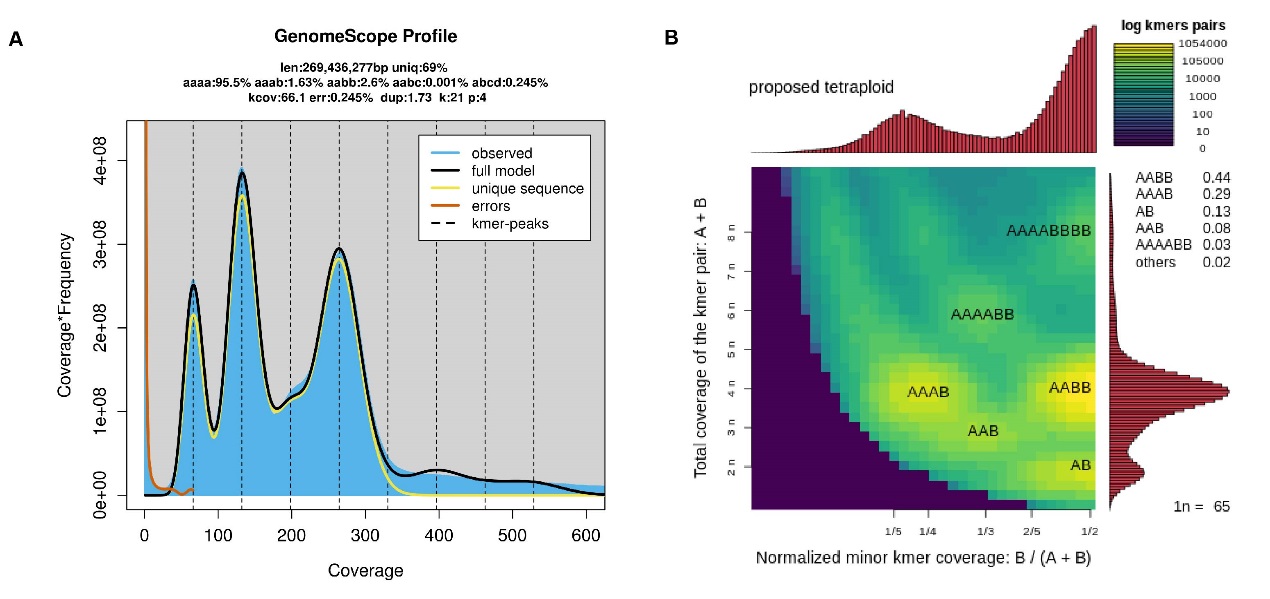


Figure S7. Ploidy prediction using whole-genome Illumina reads by Genomescope2.0 (A) and Smudgeplot (B).


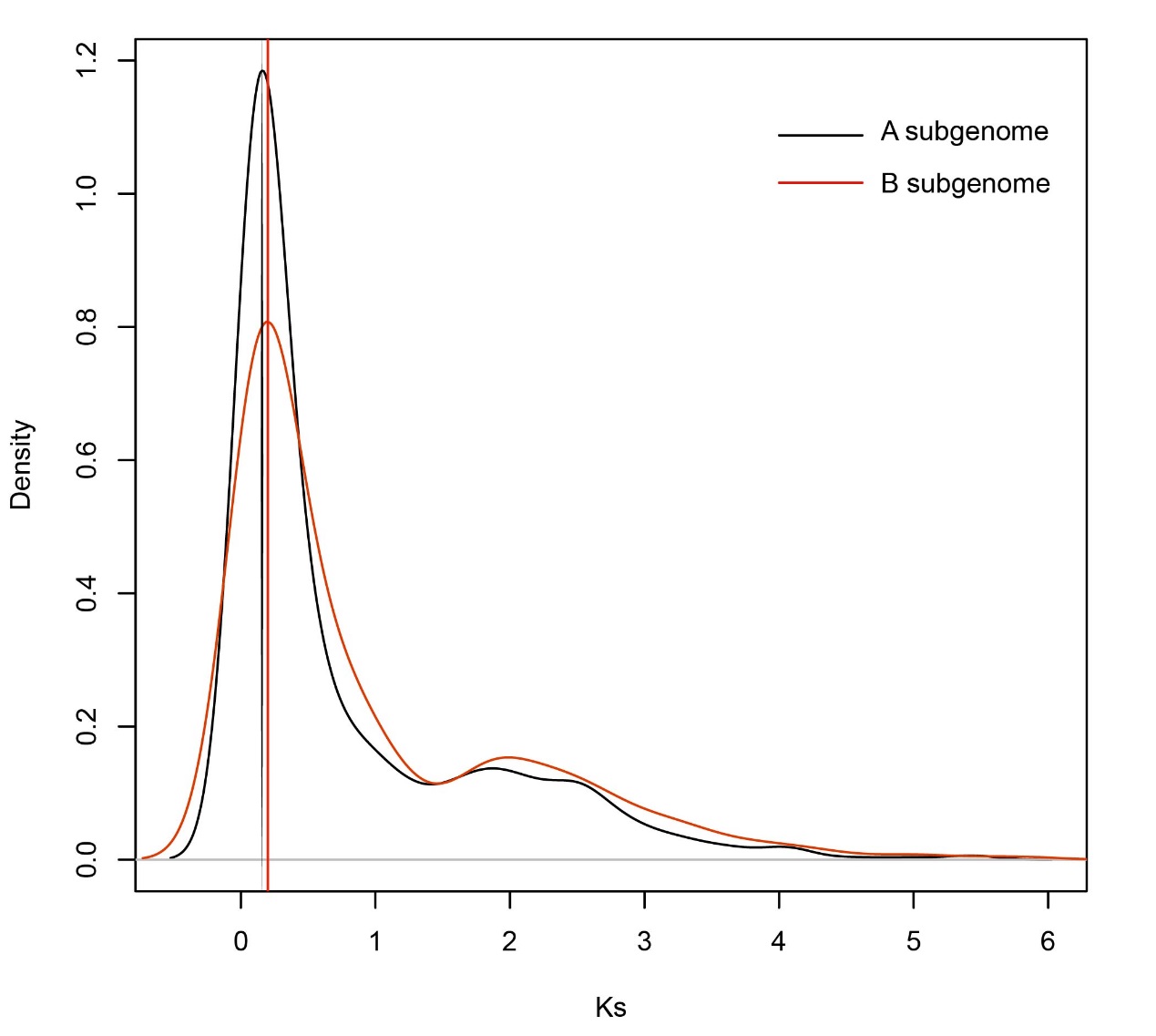


Figure S8. Two subgenomes in tetraploid species were assigned based on the divergence time with diploid *C. rotundifolia*.


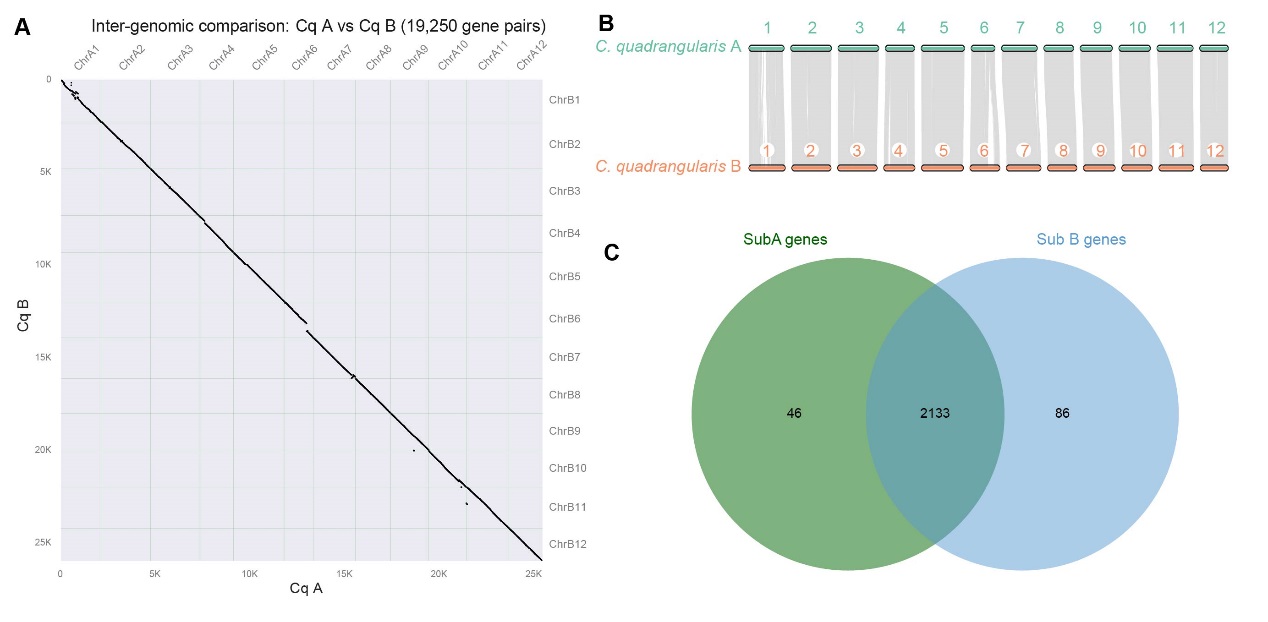


Figure S9. Collinearity relationship (A-B) and single copy genes of BUSCO (C) between two subgenomes.


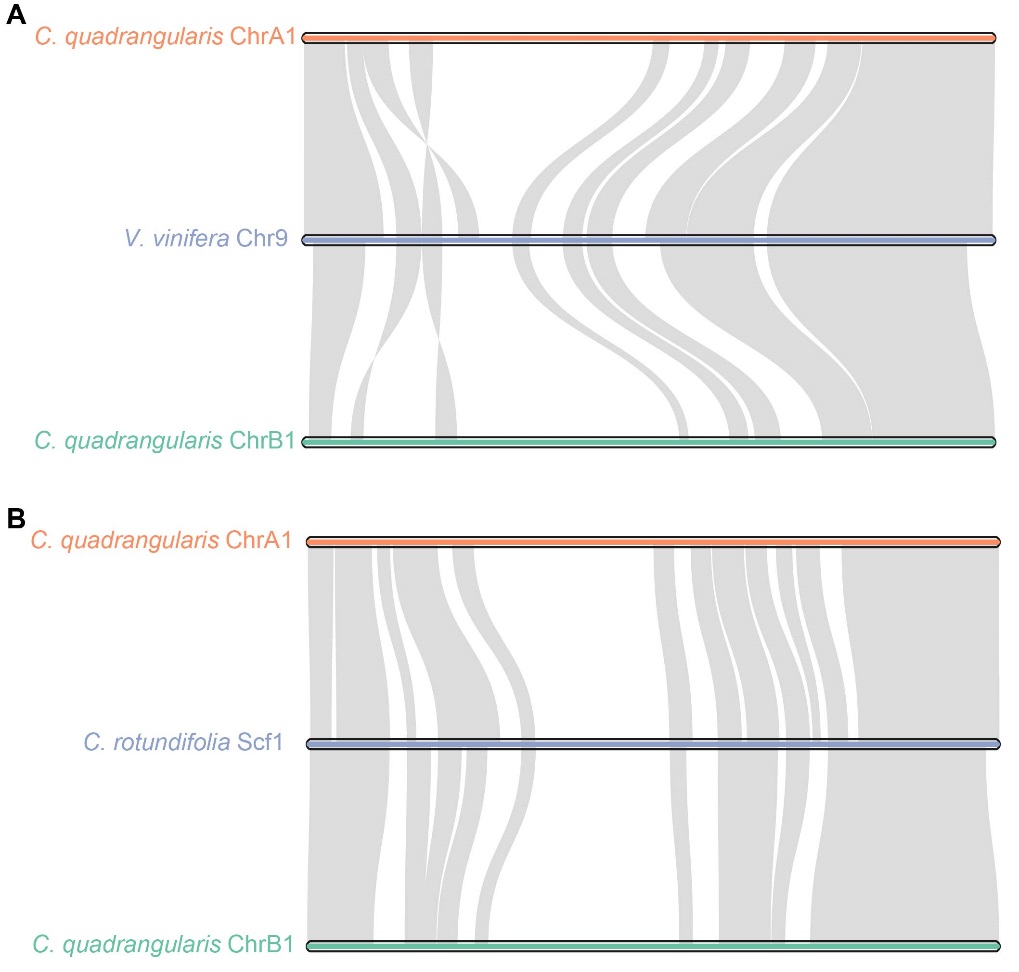


Figure S10. The collinear relationships of Chr1 pairs of *C. quadrangularis* compared to Chr9 of *V. vinifera* (A) and Scaffold1 of *C. rotundifolia* (B).
