# Supplementary material for: The Cissus quadrangularis genome reveals its adaptive features in an arid habitat
Source: Hortic Res. 2024 Feb 2;11(4):uhae038. doi: 10.1093/hr/uhae038 (PMC11001597; doi:10.1093/hr/uhae038)
Supplement: Web_Material_uhae038 [file web_material_uhae038.zip › 3.2. Supplemental Figure 11-20.docx]

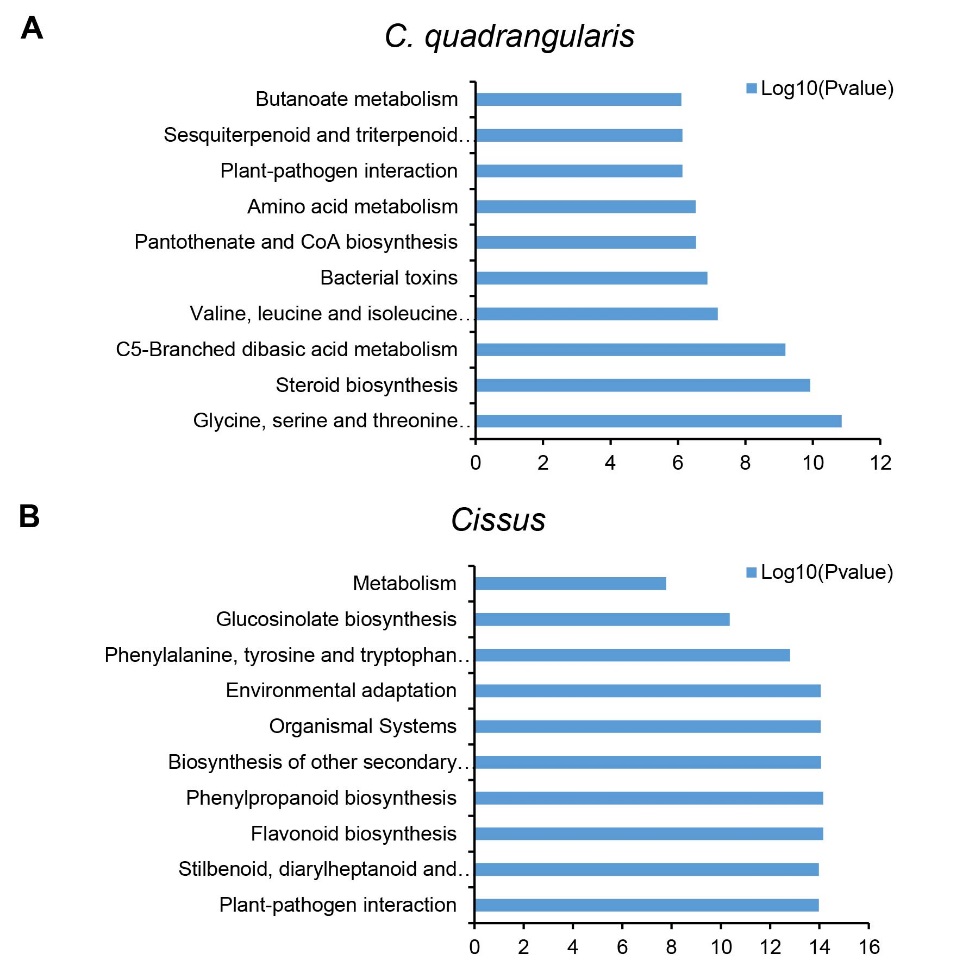


Figure S11. Functional enrichment of genes uniquely distributed in Chr1 pairs of *C. quadrangularis* and two *Cissus* species. A. KEGG enrichment of genes uniquely distributed in the Chr1 of *C. quadrangularis* when compared to Chr9 of *V. vinifera* and Scaffold1 of *C. rotundifolia*. B. KEGG enrichment of genes in Chr1 in *C. quadrangularis* and *C. rotundifolia* when compared to Chr9 of *V. vinifera*.


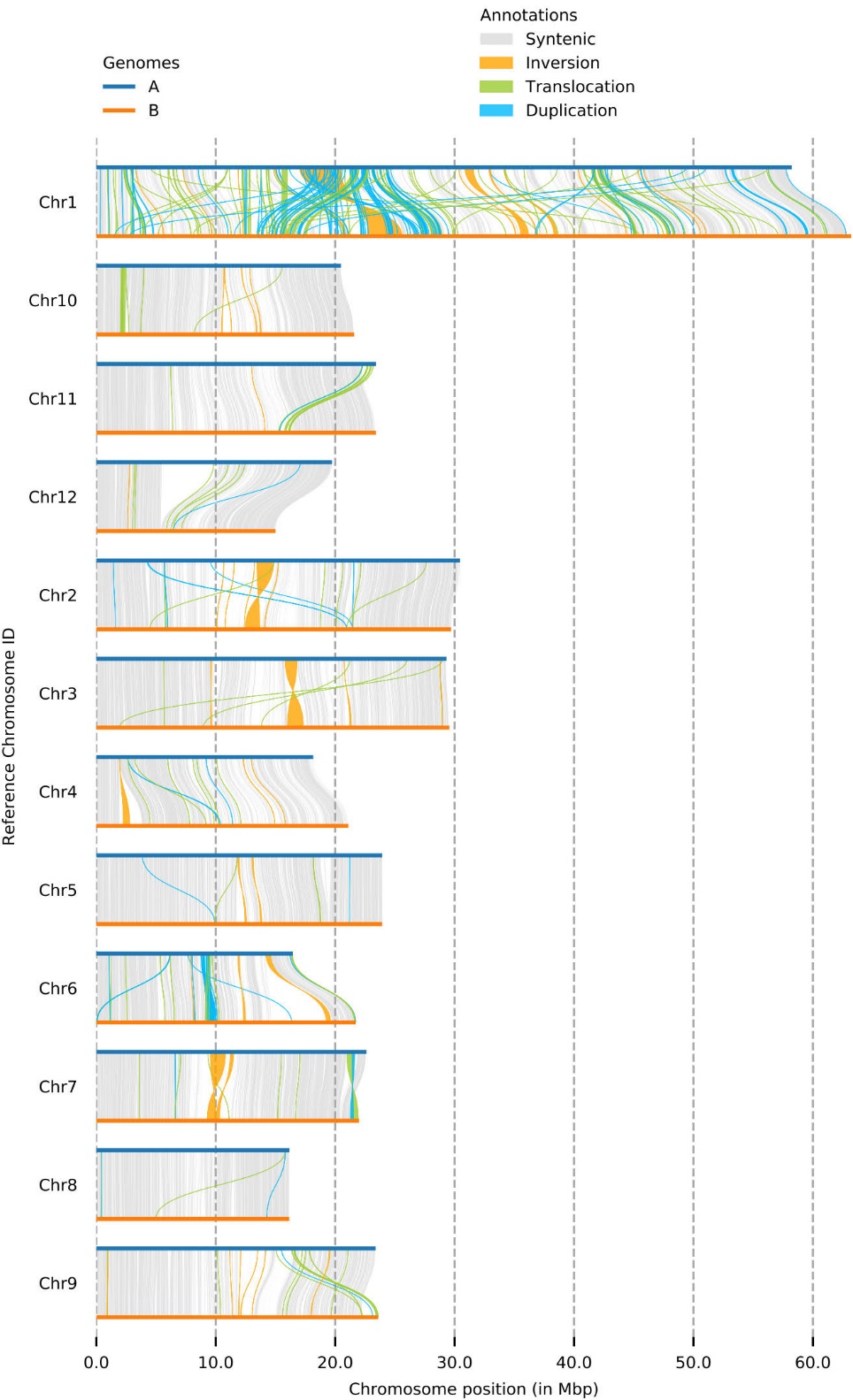


Figure S12. The identification of structural variations (SVs) between two subgenomes of *C. quadrangularis*.


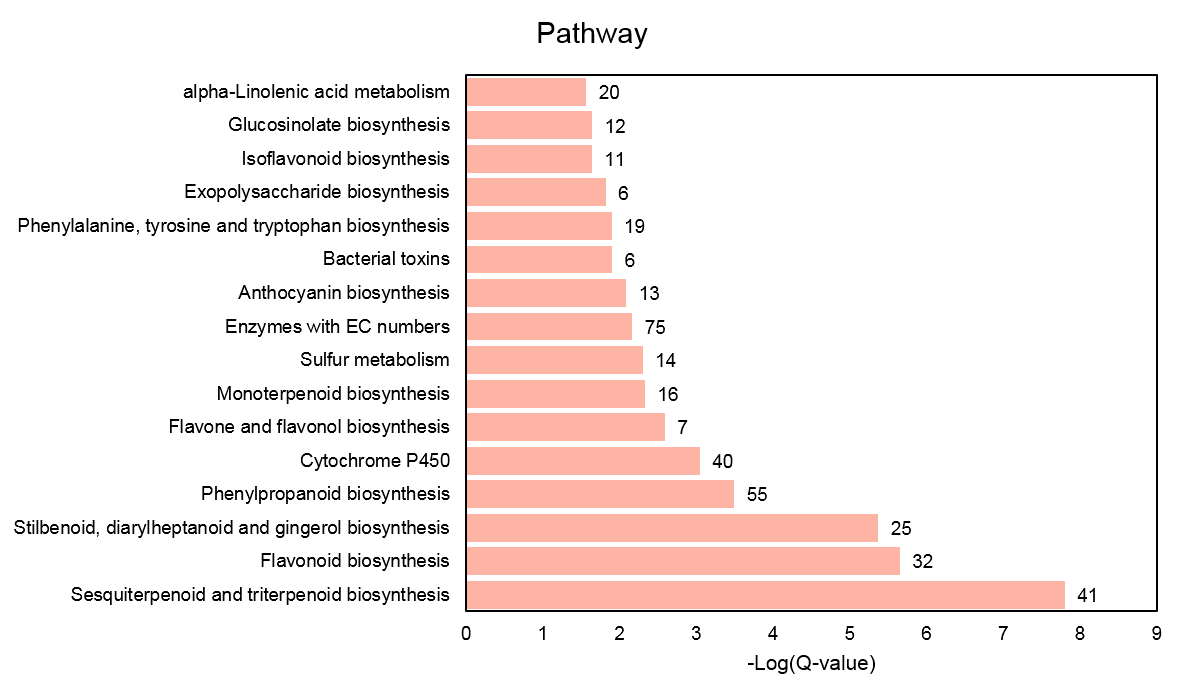


Figure S13. Functional enrichment of genes in SV regions of *C. quadrangularis*. The number on the column represents the number of genes.


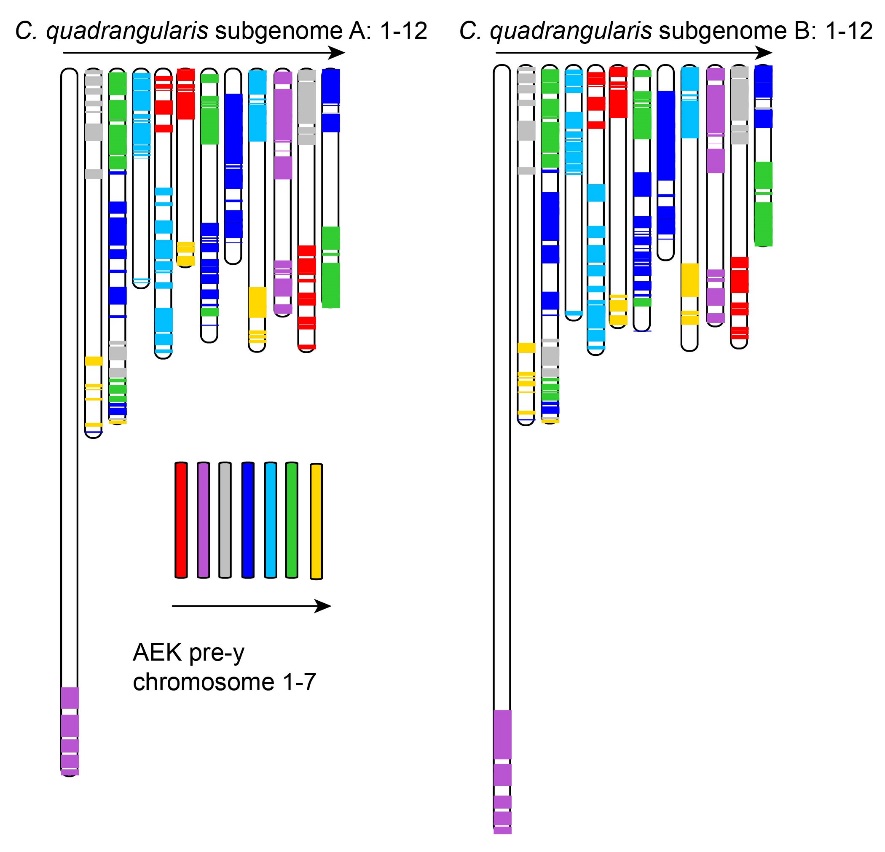


Figure S14. Gross chromosomal rearrangement underlying 12 modern chromosomes in A and B subgenome of *C. quadrangularis*.


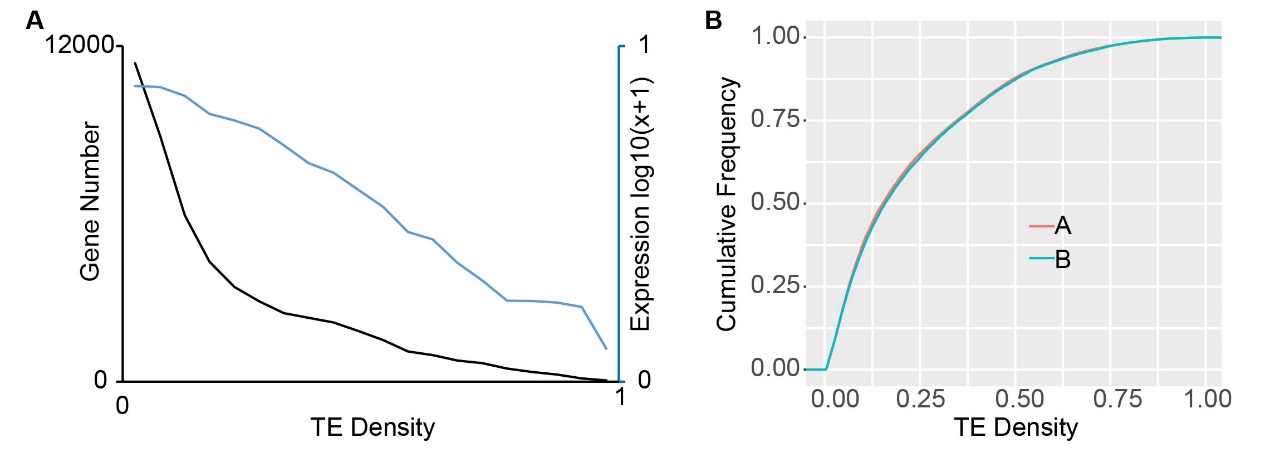


Figure S15. The impact of TE density on expression for the allotetraploid *C. quadrangularis*. A. Gene expression is negatively correlated with TE density in the genome. TE density was calculated in 5kb up- to 5kb downstream windows of the gene. The total number of genes in each of the twenty bins (5% increments in TE density) has presented in black. B. Comparison of TE densities near the genes between two subgenomes. TE densities near genes in *C. quadrangularis* subgenome were similar.


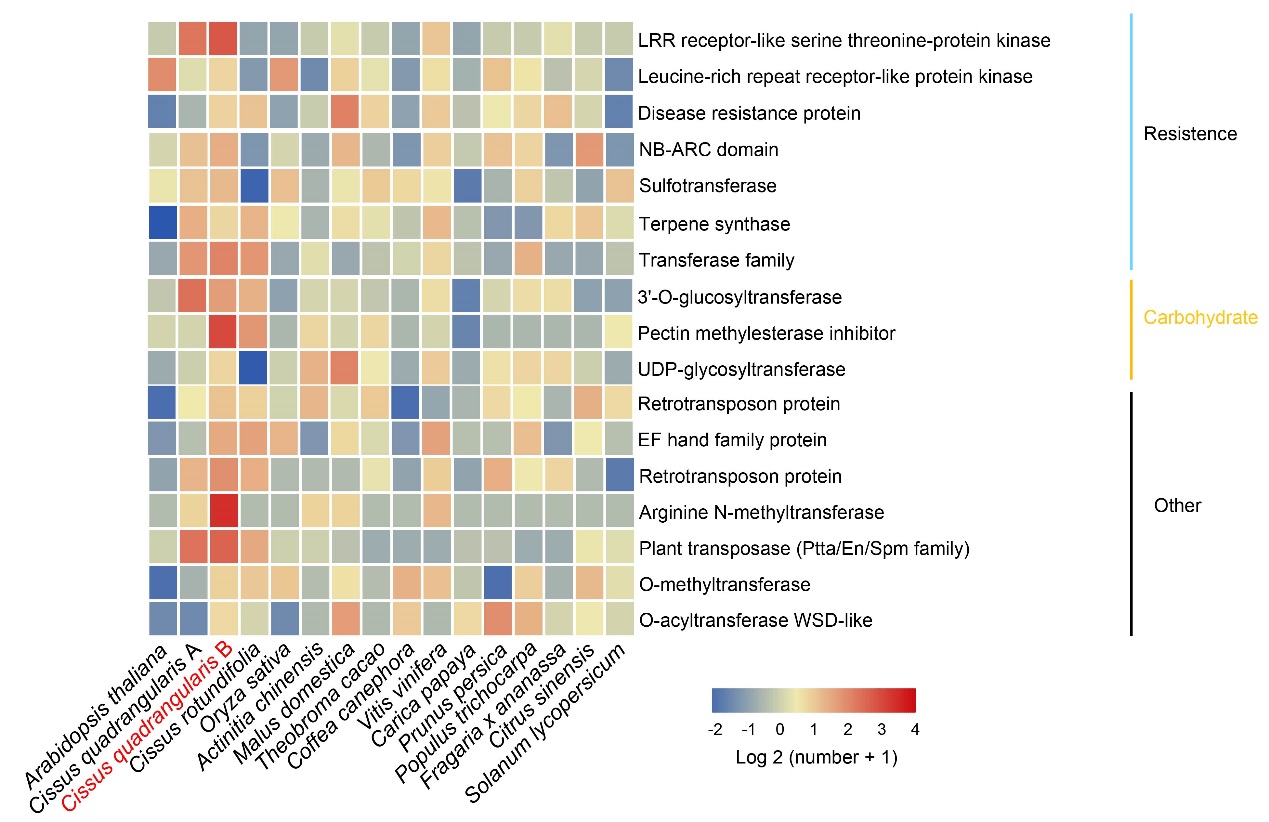


Figure S16. Heat map showing categorized orthogroups that have significantly increased paralogous numbers in the *C. quadrangularis* B subgenome compared to other angiosperms.


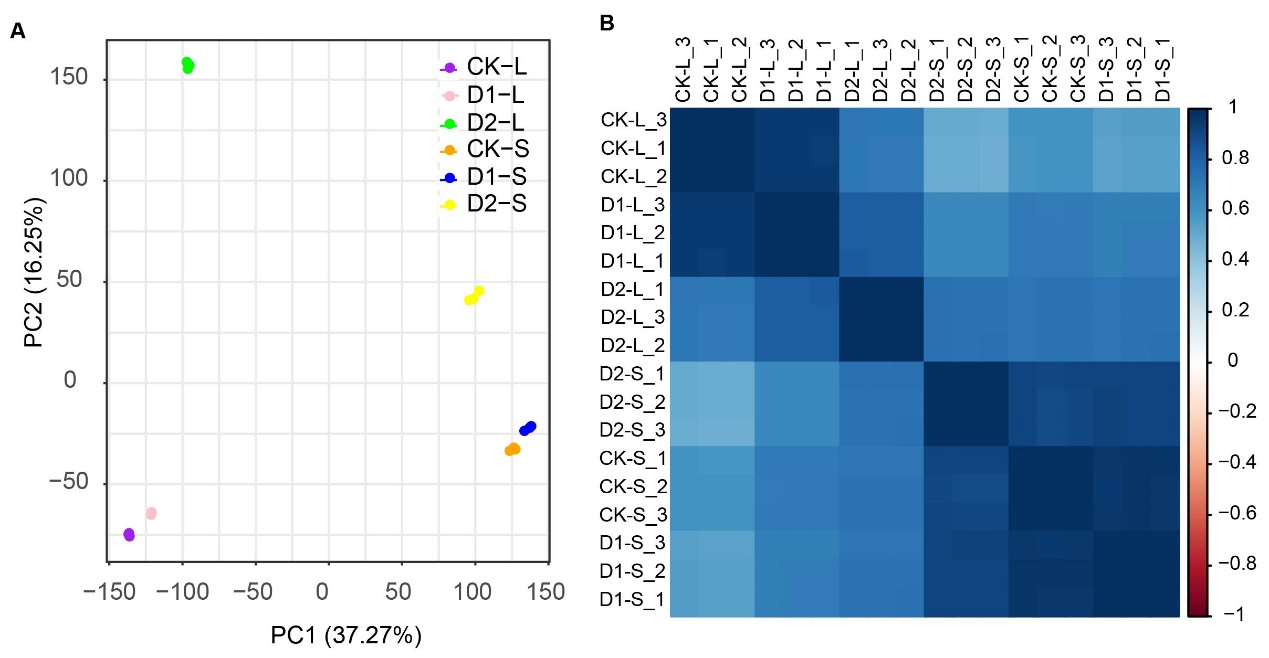


Figure S17. Data quality of the drought transcriptome. A. Principal component analysis of transcriptome of drought treatments with three replications. B. Correlation relationships in three drought periods with three replications in leaf (L) and stem (S) under drought stress. SRWC, soil relative water content; CK, 60% SRWC; D1, 30% SRWC; D2, 10% SRWC.


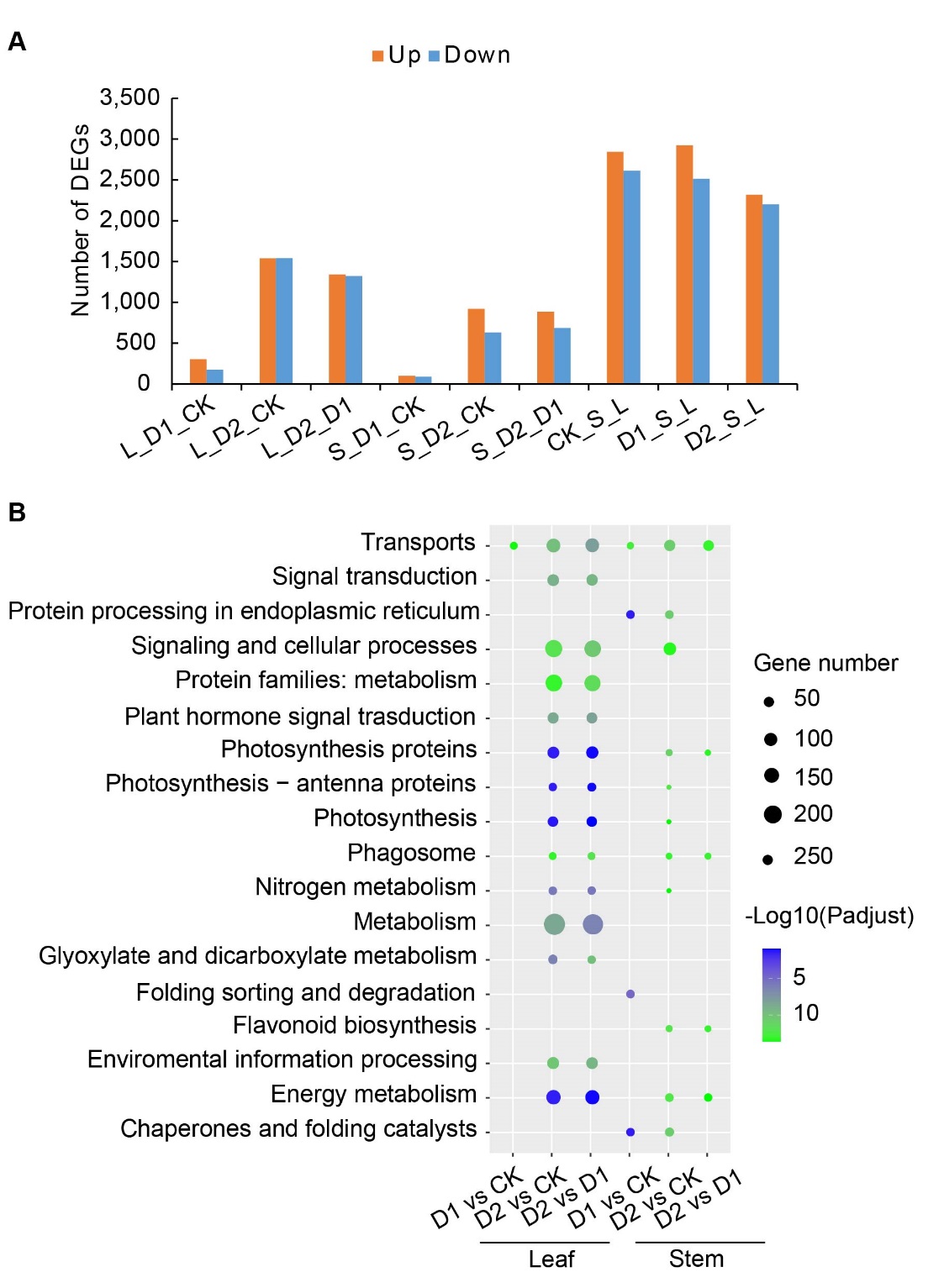


Figure S18. The dynamic changes of genes under drought. A. The number of differentially expressed genes (DEGs) among the nine comparison groups (L_D1_CK, L_D2_CK, L_D2_D1, S_D1_CK, S_D2_CK, S_D2_D1, CK_S_L, D1_S_L, D2_S_L). B. KEGG enrichment analyses of down-regulated DEGs among various comparisons for leaves and stems.


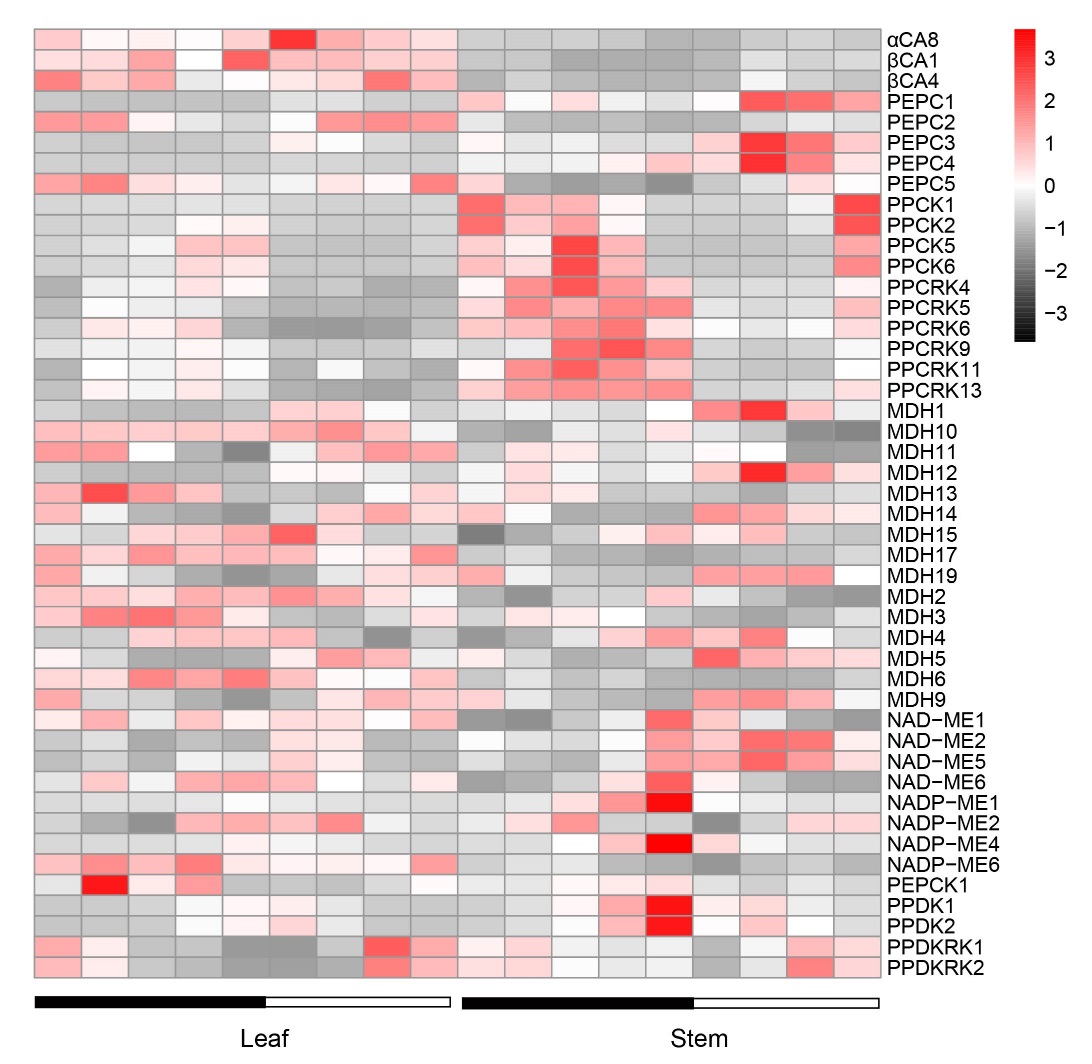


Figure S19. The gene expression of core CAM genes that have putative function in stem during a diel cycle.


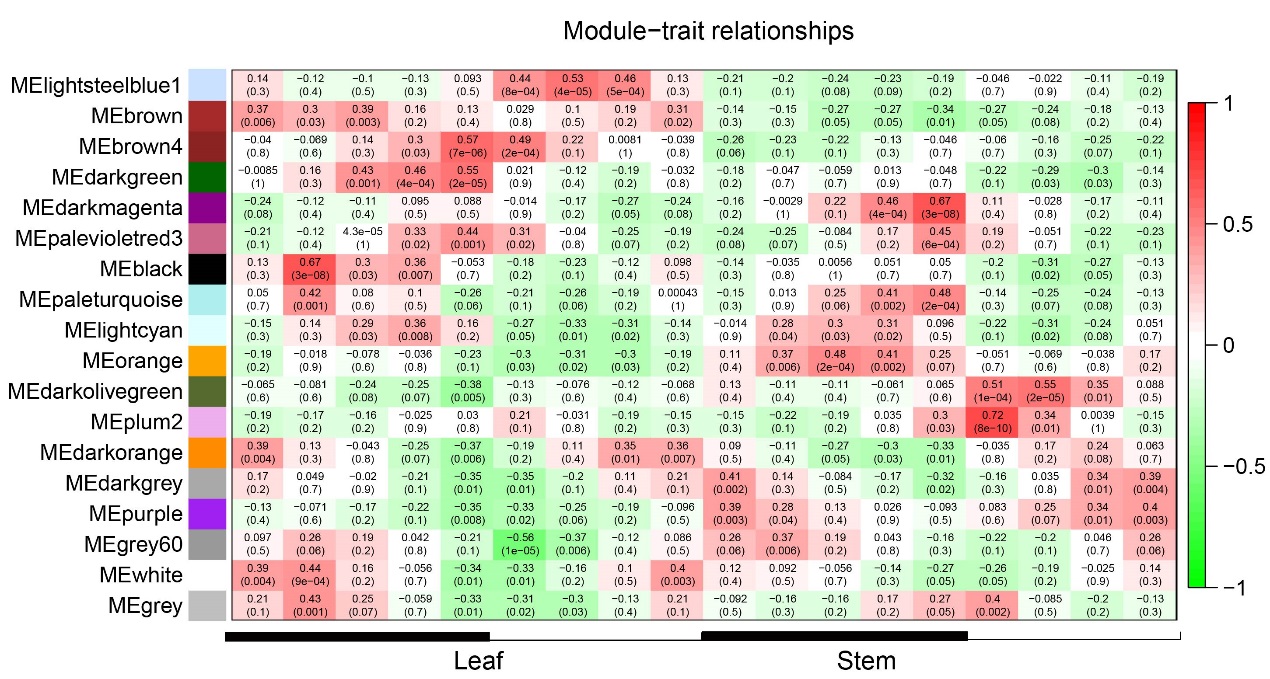


Figure S20. The module and phase relationships of diel expressed genes of the *C. quadrangularis*.
