# Supplementary material for: The Cissus quadrangularis genome reveals its adaptive features in an arid habitat
Source: Hortic Res. 2024 Feb 2;11(4):uhae038. doi: 10.1093/hr/uhae038 (PMC11001597; doi:10.1093/hr/uhae038)
Supplement: Web_Material_uhae038 [file web_material_uhae038.zip › 3.3. Supplemental Figure 21-23.docx]

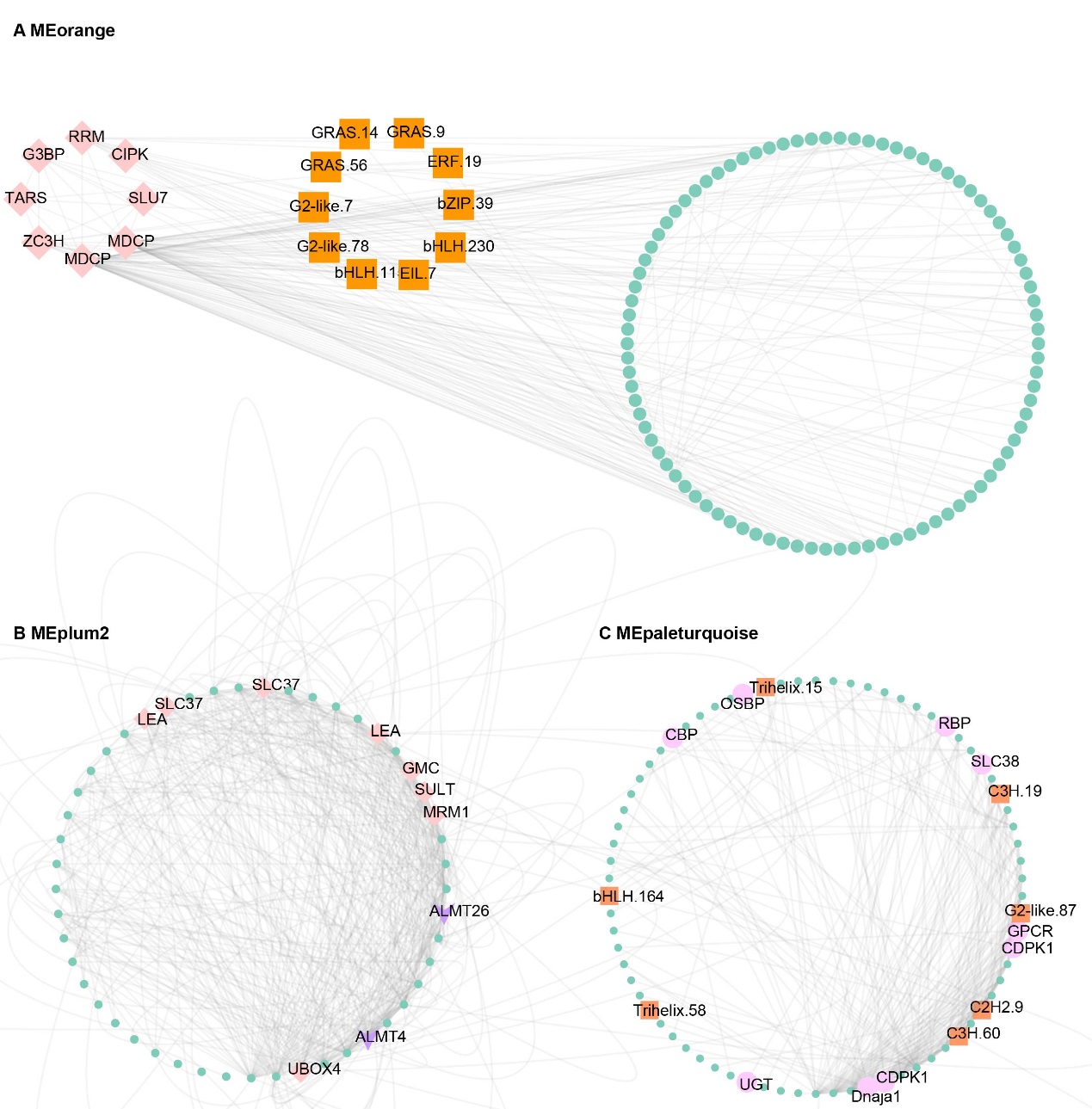


Figure S21. Three significant modules constructed by WGCNA. The orange square, pink diamond, purple triangle represented transcription factors, hub-genes, and core CAM genes in respectively module.


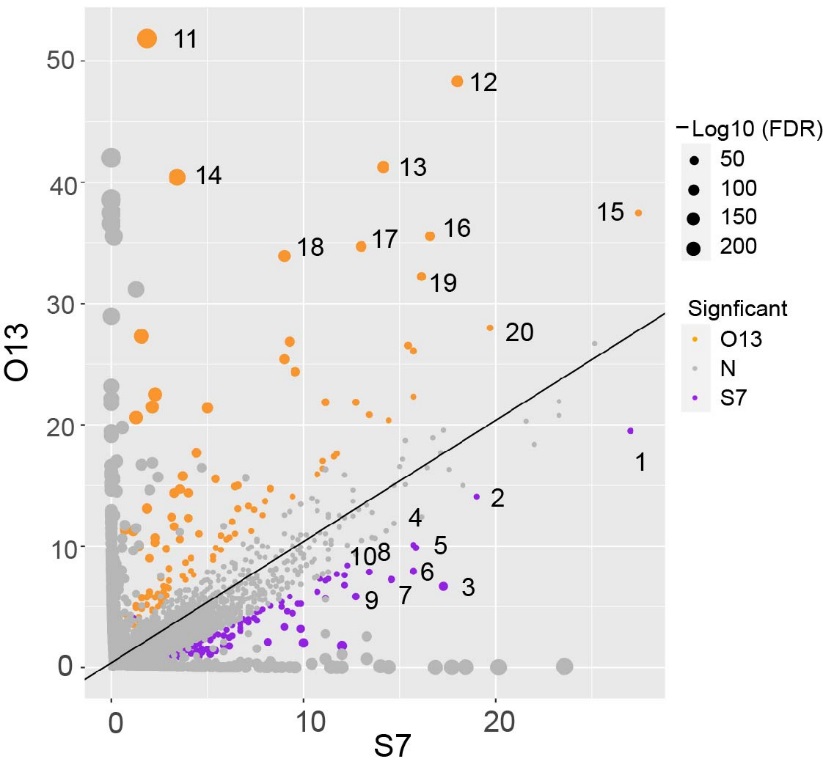


Figure S22. Scatter plot displaying the expanded orthogroups in 7 succulent plants (S7) and 13 other non-succulent plants (O13). Numbers in square brackets associated with circle sizes stand for ‒log (P-adjust). 1–20 are terpene synthase 21, expansin, plant self-incompatibility protein S1 family, type I inositol-1,4,5-trisphosphate 5-phosphatase, heat shock protein, aspartyl protease family protein, MLP-like protein 28, cytochrome P450, UDP-glycosyltransferase, sugar transporter, ribonuclease H-like superfamily protein, serine threonine-protein kinase, retrotransposon protein, LRR and NB-ARC domains-containing disease resistance protein, cysteine-rich receptor-like protein kinase, LRR receptor-like serine threonine-protein kinase, F-box family protein, receptor-like protein kinase, wall-associated kinase family protein, xylem cysteine peptidase.


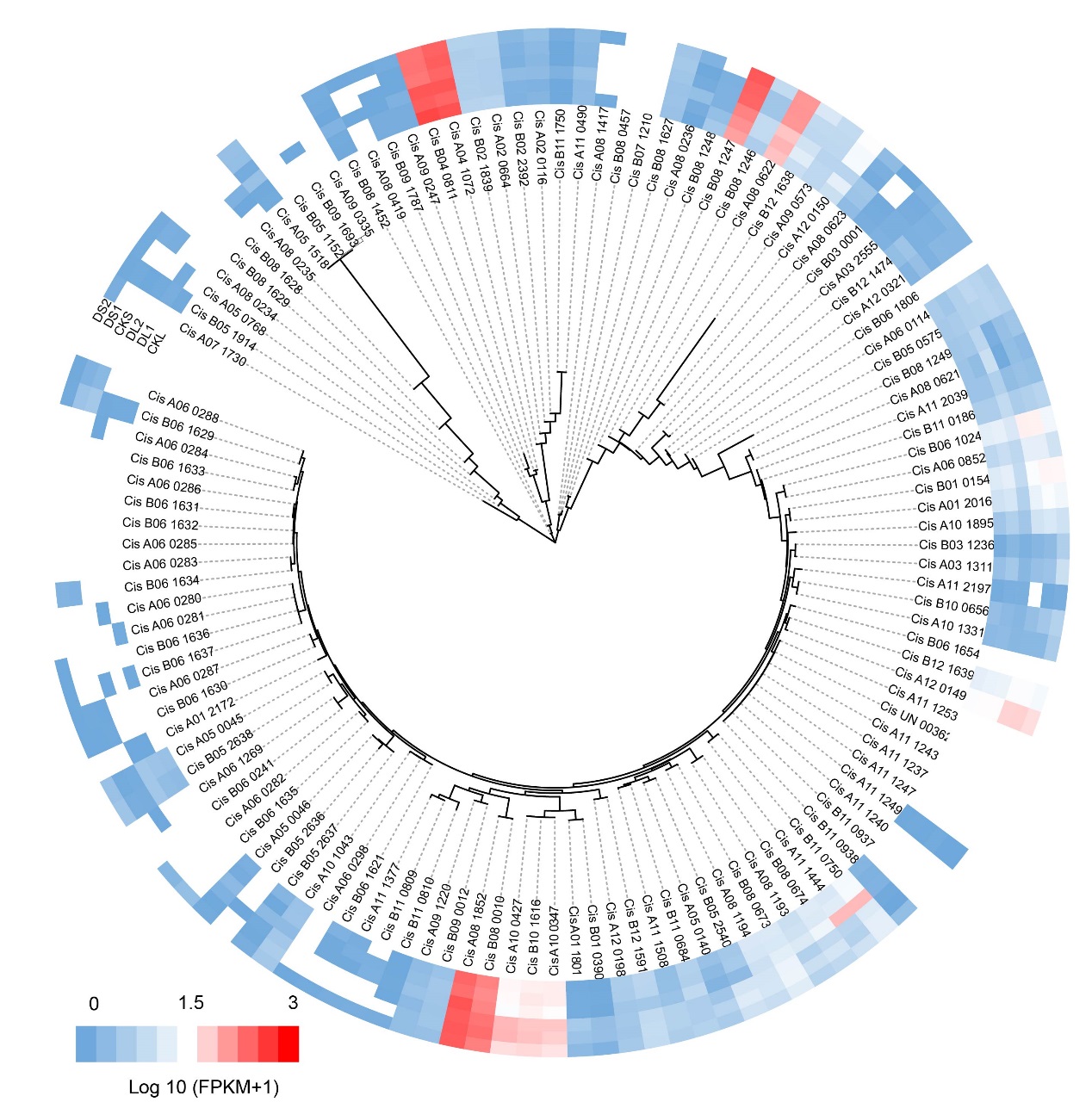


Figure S23. The gene expression of late embryogenesis abundant genes in leaves and stems of *C. quadrangularis* under drought treatments. DS1 and DS2 present 30% and 10% SRWC in stems; DL1 and DL2 present 30% and 10% SRWC in leaves; CKS and CKL represent 60% SRWC in stems and leaves, respectively.
