# Supplementary material for: The Cissus quadrangularis genome reveals its adaptive features in an arid habitat
Source: Hortic Res. 2024 Feb 2;11(4):uhae038. doi: 10.1093/hr/uhae038 (PMC11001597; doi:10.1093/hr/uhae038)
Supplement: Web_Material_uhae038 [file web_material_uhae038.zip › Figure S2,S3 & Table S7.docx]

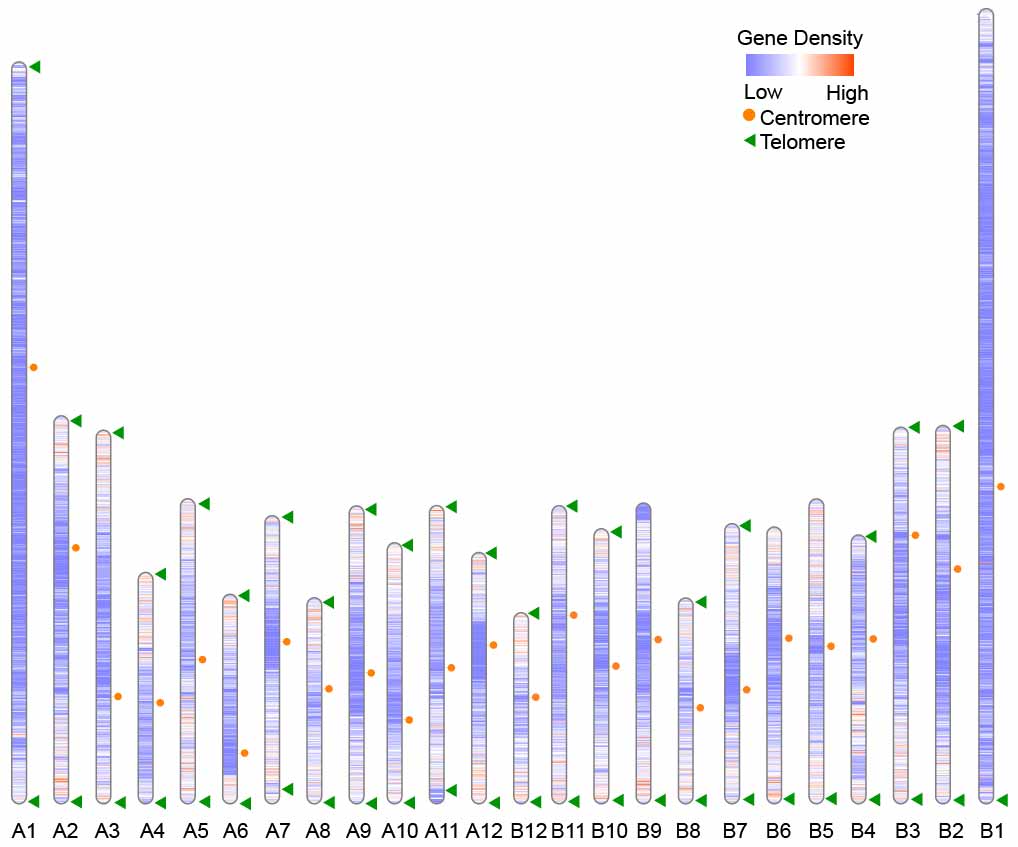


Figure S2. Visualization plant-specific seven-base telomere repeat sequence in assembly.


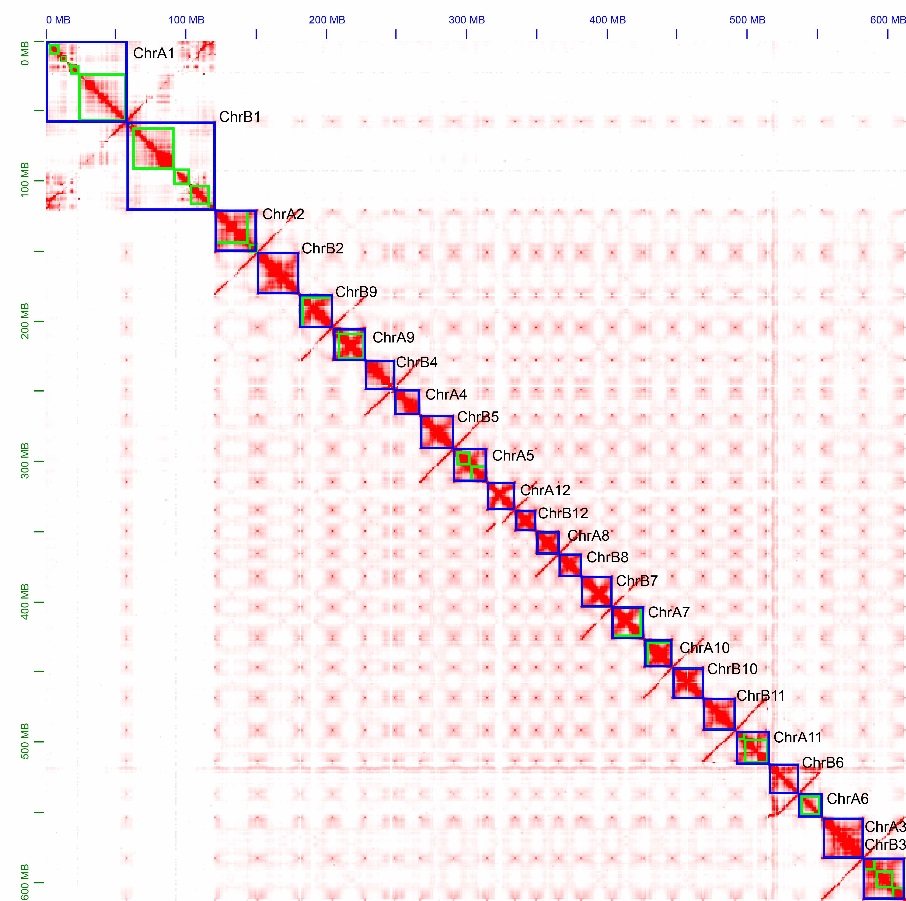


Supplemental Figure 3. The Hi-C heatmap of C. quadrangularis chromosomes during assembly process. The green boxes present contigs by HiFi sequencing and the blue boxes present pre-chromosomes after manual adjustment.

Supplemental Table 7. Genomic sequence feature statistics of *Cissus quadrangularis* (Cq)

|  | Cq | Cq A subgenome | Cq B subgenome | Other Scaffold |
| --- | --- | --- | --- | --- |
| Gene number | 51,857 | 25,053 | 25,668 | 1,136 |
| Max gene length (bp) | 75,103 | 75,103 | 73,843 | 58,648 |
| Min gene length (bp) | 168 | 168 | 180 | 180 |
| Average gene length (bp) | 4,208 | 4,226 | 4,222 | 3,486 |
| Protein number | 51,914 | 25,020 | 25,650 | 1,244 |
| Max protein length (aa) | 5,090 | 5,088 | 5,090 | 4,574 |
| Min protein length (aa) | 52 | 56 | 52 | 52 |
| Average protein length (aa) | 394 | 398 | 396 | 308 |
| Average exon length (bp) | 231 | 230 | 231 | 243 |
| Exon number | 266,665 | 130,247 | 132,064 | 4,354 |
| Intron number | 196,188 | 95,330 | 97,640 | 3,218 |
| Number of single exon genes | 7,436 | 3,623 | 3,677 | 136 |
| Number of single intron genes | 8,183 | 3,869 | 3,948 | 366 |
